# A Systematic Review and Meta-Analysis on the Global Seroprevalence of Porcine Reproductive and Respiratory Virus (PRRSV) in Pigs and Wild Boars: A Widespread and Impactful Swine Virus

**DOI:** 10.3390/vetsci13030304

**Published:** 2026-03-23

**Authors:** Giulia Graziosi, Consiglia Longobardi, Caterina Lupini, Elena Catelli, Gianmarco Ferrara

**Affiliations:** 1 Department of Veterinary Medical Sciences, University of Bologna, 40064 Ozzano dell’Emilia, Italy; caterina.lupini@unibo.it (C.L.); elena.catelli@unibo.it (E.C.); 2 Department of Veterinary Medicine and Animal Production, University of Naples, Federico II, 80137 Naples, Italy; consiglia.longobardi@unina.it; 3 Department of Veterinary Sciences, University of Messina, 98168 Messina, Italy

**Keywords:** porcine reproductive and respiratory syndrome, PRRSV, swine, wild boars

## Abstract

Porcine reproductive and respiratory virus (PRRSV) has a significant clinical and economic impact on pig farming. The purpose of this study was to assess the global seroprevalence of PRRSV in pigs and wild boars using a systematic review and meta-analysis approach. Following the Preferred Reporting Items for Systematic Reviews and Meta-analyses (PRISMA) guidelines, a systematic review and meta-analysis on global serological data of PRRSV in pigs and wild boars was conducted. Studies published between 1993 and 2025 were searched in three electronic databases, including PubMed, Web of Science, and Scopus. A total of 86 publications, belonging to 41 countries and including 690,771 animals, were deemed eligible. Following the identification and removal of outlier studies, the pooled serological prevalence was 14% (95% CI: 9–19%), and a high between-study heterogeneity was detected (*I*^2^ = 99.9%, *p* < 0.000001). Subgroup analyses showed statistically significant differences according to continents, with the highest prevalence found in Asia (P: 29%, 95% CI: 16–43%), and species, with a higher prevalence in domestic pigs (P: 26%, 95% CI: 18–35%) than in wild boars (P: 2%, 95% CI: 1–3%). Overall, the information hereby presented provides an overview of the global PRRSV situation and identifies key factors associated with increased prevalence, primarily related to animal density. These insights could inform future surveillance strategies and help target interventions to mitigate the disease burden and safeguard swine health.

## Simple Summary

Porcine reproductive and respiratory virus (PRRSV) is a widespread and impactful virus of swine. By conducting a systematic review and meta-analysis, this study summarizes global serological data on PRRSV in pigs and wild boars. The results obtained underscore a high disease burden in domestic pigs, especially in some regions of the world, and identify key factors associated with increased prevalence.

## 1. Introduction

Porcine reproductive and respiratory syndrome (PRRS) is considered one of the most impactful diseases of the swine industry, characterized by reproductive disorders in sows and respiratory dysfunction in pigs of all ages. First documented in the United States in 1987, porcine reproductive and respiratory syndrome virus (PRRSV) was subsequently reported in Japan in 1989, and in Germany in 1990, and, within a few years, it spread rapidly worldwide [1]. Nowadays, the virus has been reported in all countries, and pigs, including wild swine, are recognized as the only natural host [2]. The causal agent is an RNA virus that belongs to the *Arteriviridae* family, *Variarterivirinae* subfamily, in the order *Nidovirales* [3]. The genome is around 15 kb in length and includes at least 10 open reading frames (ORFs) that encode envelope glycoproteins (GP2–GP5), membrane protein M, nucleocapsid protein N, and several non-structural proteins (14, including RNA replicase) [4]. PRRSV, like other RNA viruses, evolves rapidly, mainly due to genetic drift and pressure (particularly those carried out by host immunity), resulting in the genetic, antigenic, and biological variability of circulating strains [5]. This property is due to the absence of 3’ proofreading ability in RNA-dependent RNA polymerase, leading to error-prone replication. The high mutation rate complicates the taxonomy of this virus. As an example, two distinct species are described according to the most recent virus taxonomy, *Betaarterivirus suid 1* and *Betaarterivirus suid 2*, which share up to 70% nucleotide identity and have been classified within two different subgenera, *Eurpobartevirus* and *Ampobartevirus*, respectively [3]. Previously, these were referred to as PRRSV-1 and PRRSV-2, which are historically distinct, with the former originating in Europe and the latter in North America [6]. Other strains have also been described (sometimes as coinfection), such as the highly pathogenic PRRSV (HP-PRRSV) strain, which has caused outbreaks in China, and the recombinant strains GM2 and NADC30-like (resulting from a recombination between HP-PRRSV and North American strains), which have been prevalent in some areas [7]. Genome recombination events, which mostly affect ORF5 and its encoding protein GP5, as proven by whole genome sequencing studies, can influence strain virulence and reduce vaccine efficacy, leading to immune escape [8]. The detailed study of this sequence has led to the classification into lineages and sublineages [9,10]. PRRSV type 2 has been documented in North America and Asia, with distinct lineages. Within the seven North American lineages, introductions into Asian and European nations were common, with some leading to impactful outbreaks [11]. PRRSV type 1 has been reported in Eastern Europe (Poland, Belarus, Lithuania, and Russia). Furthermore, whereas cross-border transmission is common in Western Europe, a clear geographical boundary was found at the eastern Polish border (Western Europe). Type 1 PRRSVs have been imported into five non-European countries: the United States, South Korea, Canada, Thailand, and others [11]. The other variants have been described mainly in Asian countries. As a result of its spread, PRRSV is routinely detected in worldwide swine populations by serological and genetic studies.

Large-scale studies have shown molecular prevalence rates of approximately 20–30% of tested pigs. These percentages increase significantly during outbreaks. Furthermore, several studies have highlighted how different risk factors are correlated to higher molecular or serological prevalences, such as, for example, seasons (higher prevalence has been described during winter and spring), or pig density and population size [12]. Furthermore, the introduction of gilts was identified as one of the most important causes of PRRS outbreaks, including the number of access points to barns, the number of outgoing movements of pigs, and environmental factors [13].

From a clinical point of view, PRRSV is a highly infectious swine virus that causes reproductive failure (late-term abortion, stillbirth, and mummified birth) in sows, reproductive disorders in boars, and respiratory symptoms (dyspnea, coughing, and wheezing) in pigs of all ages [14]. PRRSV-related reproductive problems are caused by viral damage to the placenta and endometrium. Reduced growth of piglets has been described in farms where PRRSV is circulating [15]. Generally, PRRSV-1 strains are linked with a significant morbidity and death rate, predominantly causing fever, haemorrhagic disease, severe lung damage, and multiorgan failure [16,17]. In contrast, PRRSV-2 exhibits various pathogenicities, genetic diversity, and a cyclical pattern of severe manifestations. Clinical symptoms and infection type (subclinical, acute, or chronic) are determined not only by the infecting strain but also by growth stage, immunological status, the presence of secondary or co-infecting pathogens, and environmental factors. PRRSV coinfection viruses often include porcine circovirus 2 (PCV2), pseudorabies virus (PRV), porcine parvovirus (PPV), porcine respiratory coronavirus (PRCoV), classical swine fever virus (CSFV), and swine influenza virus (SIV). Moreover, coinfections with bacteria such as *Haemophilus parasuis*, *Mycoplasma hyopneumoniae*, *Streptococcus suis*, *Bordetella bronchiseptica*, and *Actinobacillus pleuropneumoniae* are also frequent [18].

The virus is shed by many body fluids (saliva, mucus, serum, urine, feces, milk, semen, and vaginal fluids) and is directly transmitted via the respiratory and sexual routes, including artificial insemination [1]. Also known as “porcine blue ear disease”, PRRSV owes its pathogenicity to the modulation of the immune system, resulting in immunosuppressive effects. In fact, PRRSV can replicate in monocyte/macrophages, causing functional disorders, apoptosis, and lymphoid depletion [19]. Moreover, PRRSV is able to impair many innate pro- and anti-inflammatory cytokine productions as well as interferon release, delaying the formation of neutralizing antibodies and malfunctioning natural killer cells, promoting a “persistent” viremia.

PRRSV significantly impacts the healthy development of the pig industry and is considered one of the most economically significant swine viruses [20,21,22]. The devastating economic impact has been quantified at $664 million annually, or $1.8 million a day in the USA [23]. Since PRRSV continues to pose a considerable expense to the swine industry, many approaches have been investigated over the last 20 years [22]. PRRSV control has mostly focused on biosecurity and vaccination measures [24]. A range of vaccine technologies has been developed over the years, but to date, only modified live virus and inactivated virus vaccines are commercially available [25]. Nonetheless, their efficacy can be compromised by the emergence of novel strains [26,27]. Biosecurity is thus an essential component of any PRRS control strategy, lowering the likelihood of new strain introductions and subsequent outbreaks [28]. Despite these efforts, numerous studies have shown that PRRSV continues to circulate and evolve across continents, countries, and even within the same swine operation over time [5,22]. Considering that a synthesis of global data on this topic remains unavailable, the aim of this study was to assess the global seroprevalence of PRRSV in suids by reviewing the articles in the literature and identifying any influential variables on the obtained estimate.

## 2. Materials and Methods

### 2.1. Protocol

The study protocol was developed following the Preferred Reporting Items for Systematic Reviews and Meta-Analyses Protocols (PRISMA-P) [29] and the PRISMA 2020 Statement recommendations [30] (Supporting Information S1). If any deviation from the protocol occurred, this was included in the relevant section of the document.

### 2.2. Information Sources and Search Strategy

A literature search for serological studies on PRRSV in swine and wild boars was conducted from 16 November 2024 to 16 June 2025. Three electronic databases were accessed, including PubMed (https://pubmed.ncbi.nlm.nih.gov, accessed on 16 November 2024, 14 March 2025 and 16 June 2025), Scopus (https://www.scopus.com/, accessed on 16 November 2024, 14 March 2025 and 16 June 2025), and the Web of Science core collection database (https://www.webofscience.com/wos/woscc/basic-search, accessed on 16 November 2024, 14 March 2025 and 16 June 2025). The CoCoPop mnemonic (condition, context, and population) was used to define the eligibility for papers to be included in this review (Table 1) [31].

Based on CoCoPop, the search strategy included the following concept: ‘porcine reproductive and respiratory syndrome’ AND ‘antibody’ AND ‘wild boars’ OR ‘swine’ (Table 2). Filters on language (English) and timespan (studies published after 1992) were used. Manual screening of citations and reference lists of the articles retrieved was also performed to increase the chance of finding relevant publications [32].

### 2.3. Selection Criteria

Four independent investigators (G.G., C.L., Ca.L., E.C.) screened the retrieved articles using the web-based application RAYYAN (https://www.rayyan.ai/, last accessed on 29 September 2025), following importations of search results. After duplicate removal, the concordance between the reviewers was evaluated by screening 100 randomly selected papers. This calibration phase enabled discussion and solved disagreements before the actual screening [33]. Titles and abstracts were therefore screened to exclude non-relevant articles with respect to PICO (i.e., studies under controlled conditions in which animals are deliberately exposed to PRRSV or to evaluate vaccine efficacy, immunogenicity, or other interventions). After this, the full text of the articles that passed the initial screening was downloaded and assessed for eligibility, data analysis, and extraction. An article was considered eligible if the following requirements were met: (1) the study reported information on the prevalence of PRRSV in domestic pigs or wild boars; (2) a serological test was used to assess exposure to PRRSV. Whenever the same population was surveyed in multiple publications, the article with the most exhaustive information was considered. Disagreements were resolved by consulting an experienced author in the infectious diseases field (G.F.). Reasons for study exclusion were recorded and discussed among reviewers. When needed, corresponding authors were contacted to supplement additional information.

### 2.4. Data Management and Pre-Processing

The following information was recorded and included in a data extraction sheet (Microsoft Excel 2021, version 16.49): first author, year of the publication, title, country, continent, sampling period, host, age, sex, number of animals sampled, number of animals testing positive, and diagnostic method applied. Whenever prevalence was provided in percentage format, raw numbers were obtained using integer conversion.

### 2.5. Quality Assessment

The Newcastle-Ottawa Scale (NOS) was independently used by G.F. and G.G. to assess the quality of the studies included in this investigation [34]. This scale consists of five distinct criteria, each with a possible score of up to two points: a ‘yes’ scores two, a ‘no’ receives zero, and uncertain outcomes receive one. The five aspects include whether the study aim is clear, if the detection technique is clear, the clarity of the sample site, the clarity of the sampling process (sample size calculation and random selection), and the inclusion of various risk variables (e.g., sex, age classes, and description of housing settings, where available). Studies were assigned an overall score ranging from 0 to 10 points. Papers scored ≥4 were considered eligible to be included in the analysis.

### 2.6. Statistical Analysis

Statistical analyses were performed in R software (v. 4.0.0). using the *metafor* (v. 1.9-8), *meta* (v. 8.2-1), and *dmetar* (v. 0.1.0) packages. As a first step, outliers’ identification was conducted using leave-one-out analysis and Baujat plots [35]. The serological prevalence estimate of PRRSV and its 95% confidence interval (95% CI) was then calculated using a double-arcsine transformation of data and a random-effects model [35]. The between-study heterogeneity was quantified using the Cochran’s Q and the inconsistency index (*I*^2^) of the pooled estimate, with thresholds defined as small (<25%), medium (25–50%), and large (>75%) [36].

Subgroup analysis was then planned to explore the potential sources of variability across studies [37,38]. The following variables were considered: continent where the study was conducted, decade when the study was conducted (I: 1993–2003; II: 2004–2014; III: 2015–2025), species tested (domestic pig, wild boar), serological method used, and housing of animals (free-range or farmed, for wild boars only). Whenever a study period spanned more than 10 years, the decade that included the majority of those years was assigned (e.g., study period 2007–2018; decade II). If the study period was not indicated, the decade was assigned based on publication year. To further quantify the contribution of several categorical and continuous variables to the between-study heterogeneity (continent where the study was conducted, species tested, serological method used, year of publication, and sample size of each study), a meta-regression was conducted [35].

## 3. Results

### 3.1. Literature Searches

**Figure 1:**
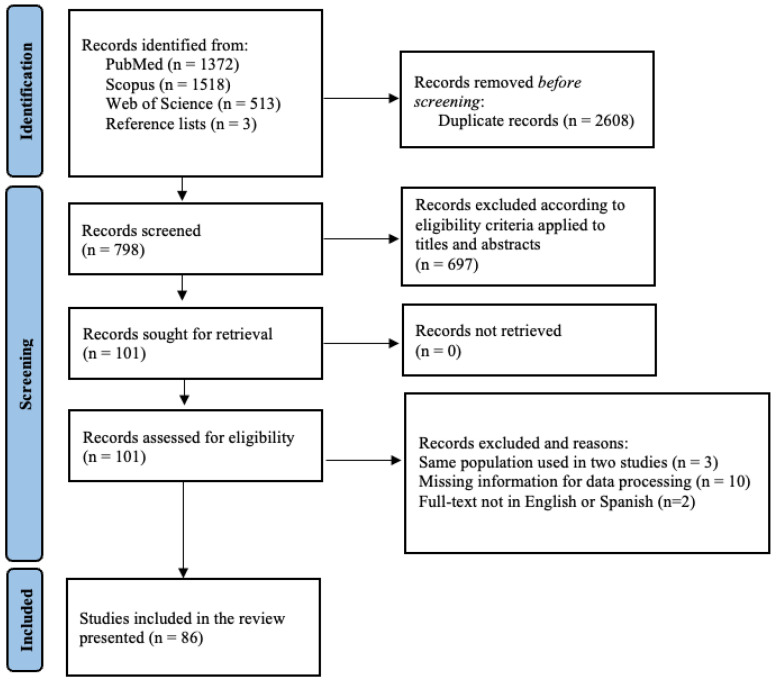
Flow diagram of the selection process of serological studies on PRRSV in swine and wild boars, identified via databases and reference lists’ reading.

The PRISMA flowchart on the selection of the eligible studies is depicted in Figure 1. A total of 3403 records were identified after the database search, 2608 were removed as duplicates, and 798 were screened for eligibility. During title and abstract screening, 697 papers were excluded as not complying with the inclusion criteria (87.3%). In the full-text assessment, 86 articles passed the full-text screening and were retained for qualitative synthesis and meta-analysis. The characteristics of the eligible studies are included in Supplementary Material S2.

With regard to geographical distribution of studies, the majority of the studies were conducted in Europe (*n* = 38), followed by Asia (*n* = 24), the Americas (*n* = 20), Africa (*n* = 3), and Oceania (*n* = 1). Particularly, most of the research was concentrated in the United States (*n* = 8), Spain (*n* = 7), Germany (*n* = 5), China (*n* = 4), and the Republic of Korea (*n* = 4). The full list of countries was included in the Supplementary Materials (Supplementary Material S2).

**Figure 2:**
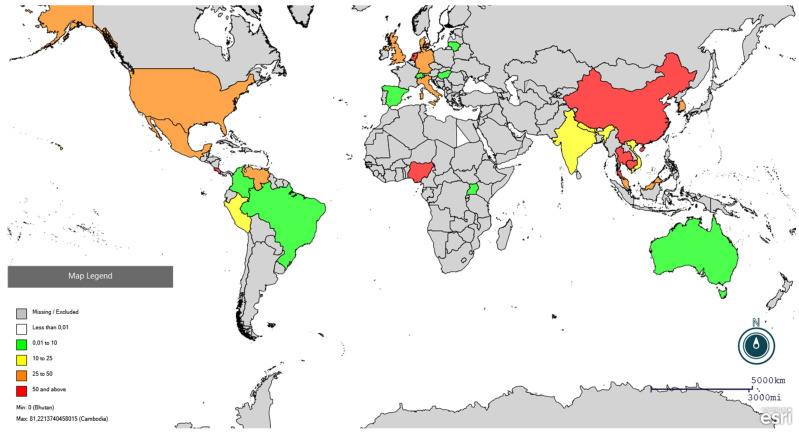
Global distribution of PRRSV seroprevalence in pigs using Epi Info online software (https://www.cdc.gov/epiinfo/index.html, accessed on 25 February 2026). Red colour indicates seroprevalence higher than 50%, orange between 25 and 50%, yellow between 10 and 25%, and green lower than 10%. Specific prevalence are present in Supplementary File S2.

**Figure 3:**
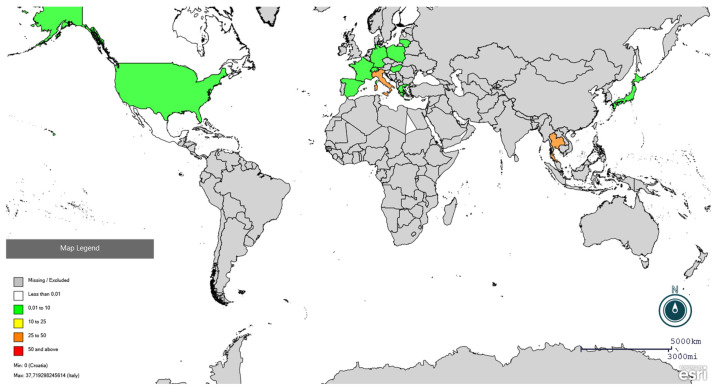
Global distribution of PRRSV seroprevalence in wild boars using Epi Info online software (https://www.cdc.gov/epiinfo/index.html, accessed on 25 February 2026). Red colour indicates seroprevalence higher than 50%, orange between 25 and 50%, yellow between 10 and 25%, and green lower than 10%. Specific prevalences are present in Supplementary File S2.

A total of 690,771 pigs and wild boars were serologically tested for PRRSV, and 207,905 individuals tested positive. The studies covered a sampling period from 1993 to 2025; 21 studies were conducted between 1993 and 2003 (I decade); 41 studies between 2004 and 2014 (II decade); and 24 between 2015 and 2025. With respect to the species tested, 50 studies out of 86 concerned domestic pigs (58%), 34 concerned wild boars (39%), and three tested both domestic pigs and wild boars (3%). Regarding the serological method used, *n* = 80 studies used the enzyme-linked immunosorbent assay (ELISA), either in-house or commercial kits, *n* = 3 used immunofluorescence antibody test (IFA), two immunoperoxidase monolayer assay (IPMA), and *n* = 1 used colloidal gold immunochromatographic assay (GICA). Only 12 studies examined age as a risk factor for the presence of anti-PRRSV antibodies (6 in wild boars and 6 in pigs), precluding any additional analysis (descriptive information is available in the Supplementary Material S2). The range of seroprevalence of PRRSV in domestic pigs and wild boars per country is shown in Figure 2 and Figure 3, respectively.

### 3.2. Quality Assessment

According to the quality assessment, all the contributions met the required standard and were therefore included in the quantitative and qualitative synthesis of results.

### 3.3. Statistical Analyses

**Figure 4:**
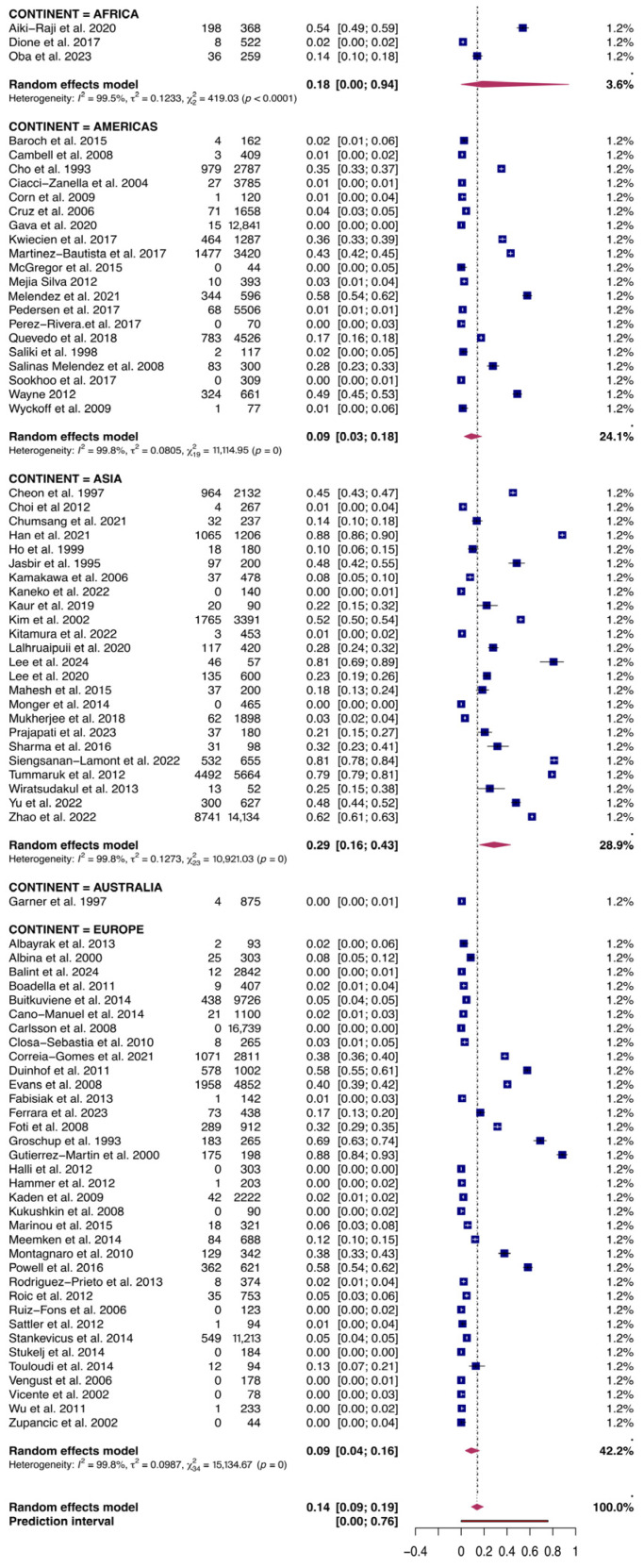
Forest plot of the random-effects meta-analysis of PRRSV serological prevalence in swine and wild boars sorted by Continent. *I*^2^ (inverse variance index), τ^2^ = the between-study variance, χ^2^, and *p*-value of the Cochran’s Q test for heterogeneity [40,42,44,45,46,47,48,49,50,51,52,53,54,55,56,57,58,59,60,61,62,63,64,65,66,67,68,69,70,71,72,73,74,75,76,77,78,79,80,81,82,83,84,85,86,87,88,89,90,91,92,93,94,95,96,97,98,99,100,101,102,103,104,105,106,107,108,109,110,111,112,113,114,115,116,117,118,119,120].

Following outlier studies identification through Baujat plot inspection and leave-one-out sensitivity analysis, three large national surveillance studies were identified as highly influential due to their large sample sizes and disproportionate impact on the pooled estimate and heterogeneity (Supplementary Material S3) [39,40,41]. After their exclusion, 135,169 animals were considered in the final meta-analysis (effective analytical sample), of which 29,535 tested positive. The estimated global pooled serological prevalence of PRRSV resulted in 14% (95% CI: 9–19%) (*I*^2^ = 99.9%, *p* < 0.001) (Figure 4). Subgroup analyses were performed according to continent, species, decade when the study was conducted, and laboratory method used (Table S1, Supplementary Material S4). Results suggested a significant difference between the pooled effect estimates for each continent (*p* < 0.0001), with the highest prevalence for Asia (P: 29%, 95% CI: 16–43%), followed by Africa (P: 18%, 95% CI: 0–94%), Europe (P: 9%, 95% CI: 4–15%), and Americas (P: 9%, 95% CI: 3–18%) having similar and much lower proportions. The species tested appears to influence the effect estimates (*p* < 0.0001), with higher prevalence in domestic pigs (P: 26%, 95% CI: 18–35%) than in wild boars (P: 2%, 95% CI: 1–3%). With respect to the sampling period covered by the studies, the highest prevalence of PRRSV was detected during the III decade (P: 25%, 95% CI: 13–40%), followed by the I (P: 15%, 95% CI: 5–30%) and II (P: 8%, 95% CI: 4–13%) decades; significant differences across decades were detected (*p* < 0.05). Lastly, the serological method used did not appear to influence the effect estimate (*p* = 0.28).

The housing, with respect to wild boars, did not appear to significantly influence the effect estimate (*p* = 0.98), with similar prevalences in free-ranging (P: 2%, 95% CI: 1–5%) and fenced animals (P: 2%, 95% CI: 0–10%) (Table S2, Supplementary Material S4). Considering the lack of data available on sex classes, defined only in 10 studies [42,43,44,45,46,47,48,49,50,51], subgroup analysis was not performed. For the age variable, inconsistencies between age identification among the obtained studies prevented comparisons.

Concerning meta-regression, the species was the only significant predictor of PRRSV seroprevalence, with wild boars showing a significantly lower odds of seropositivity compared to domestic pigs (OR = 0.69, 95% CI: 0.60–0.79, *p* < 0.0001) (Table S3, Supplementary Material S4). Covariates related to continent, publication year, sample size, and serological method used were not statistically significant (Table S3, Supplementary Material S4). Overall, the model explained 38% of the between-study heterogeneity (R^2^ = 37.57%), but residual heterogeneity remained high (*I*^2^ = 98.5%), indicating additional unmeasured factors contributing to variability.

## 4. Discussion

Porcine reproductive and respiratory syndrome is one of the most damaging and prevalent swine diseases worldwide. Consequently, practical and effective monitoring and surveillance strategies are critical for achieving full control of the infection [121]. These rely on molecular diagnostics and sequencing methods to track new variants and assess the wide genetic diversity of strains circulating worldwide, as well as on serological diagnostics, which provide data on exposure at the farm, regional, and national levels [14,122]. Serological diagnostics is, therefore, the simplest method of indirectly estimating the circulation of the virus in a population and is considered an important part of the diagnostic process. Even though a variety of assays for the detection of serum antibodies to PRRSV have been described, such as the IPMA and IFA, ELISA is the most commonly used and is considered the reference assay, particularly when population freedom must be tested or for surveillance purposes. In addition to simplicity and good diagnostic performance (especially when antigens from both PRRSV-1 and PRRSV-2 are utilized), ELISA can detect antibodies starting 9–10 days after infection and lasting at least 5–6 months [123].

In this systematic review and meta-analysis, PRRSV serological data from the literature were summarized and compared for the first time. Furthermore, our study has emphasized the global spread of PRRSV and recognized several common risk factors linked with increased prevalence. The obtained pooled seroprevalence estimate of 14% (95% CI: 9–19%) indicates a moderate exposure of suids to PRRSV, although substantial variability across studies was detected. A possible source of heterogeneity may be related to the area where the study took place, the species tested, the period, the serological method used, and other aspects related to the characteristics of the population targeted, such as housing and age classes.

According to subgroup analyses, our results showed a statistically significant association between positive PRRSV serological findings and the continent where the study took place (*p* < 0.0001); however, a lack of significance was observed for continent in the meta-regression model, which simultaneously adjusted for species, decade, and serological method used. This indicates that the apparent differences between continents in the subgroup analyses may be influenced by confounding with other study-level characteristics. Asia and Africa reported higher seroprevalence compared to those from Europe and the Americas. However, the pooled estimate for Africa should be interpreted with caution, as it was based on only three studies and showed wide confidence intervals, reflecting high uncertainty and substantial heterogeneity. PRRSV is known to spread more easily when biosecurity measures are inadequate; however, the effective implementation of farm-level biosecurity remains challenging for small-scale pig producers in Asia, as well as in many African settings, where limited resources and subsistence farming prevail [124,125]. Other factors that could explain this variability include the virulence and rate of transmission (Rt) of the different strains circulating globally (lineages and sub-lineages), which can influence the proportion of exposed animals and, consequently, those testing positive by serological assays [5]. Additionally, whether sampling was conducted during an outbreak or as part of an eradication plan, which is unknown for a great part of the studies included, could represent a source of bias for this outcome [126].

Statistical comparison among species tested revealed a significant association (*p* < 0.0001) with PRRSV serological prevalence, as confirmed by both the sub-group analysis and meta-regression. Findings indicate that PRRSV is up to 13 times more prevalent in pigs than in wild boars, suggesting a more intense circulation in domestic farm settings. In several investigations on wild boars, no seropositive animals were detected, even in regions with demonstrated circulation of the virus in pigs [52,53]. This suggests that wild boars likely play a minor role in the PRRSV epidemiology. Serological positivity in wild boars has been linked to populations living in proximity to pig farms, with limited virus spread between individuals, unlike what is observed for other infections such as African swine fever, *Brucella* spp., or tuberculosis [127,128]. A similar pattern has also been reported for other pathogens, such as swine coronaviruses, which are widespread in pigs but very rarely infect wild boars [129,130]. In addition to contact density and biosafety factors, other elements that may have influenced PRRSV prevalence are the different immune response against PRRSV in wild boar, population dynamics (such as the changes caused by African Swine Fever), and sampling deviation.

When evaluated through meta-regression, decade was not a significant predictor of PRRSV seroprevalence. The recent development of the concept of “next generation biosecurity” has led to significant improvements in the containment of infectious diseases in general [131,132]. In fact, it has been demonstrated that the application of these standards can significantly reduce the incidence of PRRSV in large pig farms, highlighting the importance of biosafety requirements [132,133]. However, given the global scale of the analysis hereby conducted, it is important to recognize that farm settings and biosecurity standards can vary widely across countries, influencing the risk of exposure to PRRSV. Next to this, the features of the primarily circulating PRRSV strains must be considered, since they have evolved to become more virulent and less pathogenic (increasing their spread), as a result of immunological pressure from seropositive/vaccinated hosts. The increasing sensitivity of modern serological diagnostic approaches could have also played a role in more effectively detecting animals exposed to the virus [134]. Lastly, it should be noted that several studies published between the 1990s and 2000s, before PRRSV became widely distributed worldwide, were specifically designed in PRRSV-free countries to demonstrate the lack of the infection. Other factors, such as age and housing (for wild boars only), showed no statistically significant association, likely due to the limited number of studies that have investigated these variables. However, the different research design of serosurveys may also affect the prevalence. Further data and analysis concerning the causal link between the years and the prevalence rates would be essential and desired. The meta-regression model explained only partially the high between-study heterogeneity (R^2^ = 35.96%), and residual heterogeneity remained high (*I*^2^ = 98.5%), indicating that additional unmeasured factors (e.g., biosecurity level, seasonal effect, farm size) may contribute to the variability in PRRSV seroprevalence.

Several limitations of this systematic review and meta-analysis were recognized. First, we acknowledge the limited geographical coverage of the eligible studies included. Although PRRSV is a widespread infection worldwide, there is little or limited published data on seroprevalence in many countries. In particular, one single study was retrieved for Oceania and a few studies for Africa; next to this, there was a complete lack of data on the exposure of pigs raised in countries in Western Asia. In some of these countries, pig farming is not practiced much for religious reasons, but pigs are still raised for minorities, resulting in viral circulation also in these districts. Similarly, published studies on wild boars’ exposure are mainly concentrated in Europe and North America, while data are completely missing from South America, Africa, South-East Asia, and Oceania. These differences can generate a misinterpretation of the actual geographic distribution of infections and overall seroprevalence, as they primarily reflect regions with more active surveillance and reporting systems. With respect to the diagnostic method applied, subgroup analysis was limited by the small number of studies using non-ELISA assays, which reduces the strength of conclusions regarding diagnostic-related differences. We also acknowledge the existence of possible research that may not have been accessible through the search strategy hereby applied, due to the exclusion of “grey literature”, as well as limitations related to language and database indexing. Governmental or industry-origin reports on PRRSV may contain relevant epidemiological information that was not captured in this review. Future efforts should promote the publication of these data in peer-reviewed journals to ensure their wider accessibility through bibliographic databases. In terms of statistical analysis, diverse statistical methods are able to quantify and adjust for publication bias in meta-analysis [135]. Nevertheless, Egger’s regression test and the trim-and-fill method do not appear to perform well in studies on proportions [136,137] and were therefore not utilized. Given the high between-study heterogeneity, publication bias may also have been incorrectly inferred [138]. A lack of studies assessing species-specific variables, such as sex or age, was noted, which limited the subgroup analysis. Future research should record these data, particularly sex and age, in a consistent format and standardized way. Furthermore, variables related to farm biosafety and biosecurity standards in pig farms were not considered due to insufficient information reported in the respective publications.

## 5. Conclusions

PPRSV is a worldwide disease that has significant effects on swine herd health and economics. This work presents the first systematic review and meta-analysis of PRRSV seroprevalence on a global scale, integrating data from studies performed over the past 35 years. Based on the results obtained, future research should aim to develop a deeper understanding of PRRSV epidemiology, particularly in countries where no reports are currently available. In addition, detailed herd-level data such as age, sex, and management practices would allow for a more accurate characterization of PRRSV transmission dynamics. Continuous surveillance is required to monitor the global spread of PRRSV and plan containment measures.

## Figures and Tables

**Table 1:** Inclusion criteria based on the CoCoPop mnemonic (condition, context, and population).

Parameter	Inclusion
Population	Domestic pigs (*Sus scrofa domesticus*) or wild boars (*Sus scrofa*); wild, free-range, or farmed.
Condition	Natural infection of PRRSV assessed through a serological test.
Context	Worldwide distribution of studies.

**Table 2:** Search lines used for the literature research of PRRSV serological studies in swine and wild boars present in PubMed, Scopus, and Web of Science databases. Boolean operators ‘OR’ and ‘AND’ were applied. In Scopus’ search line, the wildcard (*) was used to capture multiple word endings. The number of scientific contributions retrieved before duplicate removal is reported.

Database	Search Line	No. of Studies Retrieved
PubMed	((“porcine reproductive and respiratory syndrome”[Title/Abstract] OR PRRSV[Title/Abstract] OR “porcine reproductive and respiratory syndrome virus”[All Fields]) OR (“arterivirus infections”[MeSH Terms] OR “arteriviridae”[MeSH Terms])) AND (“serologic tests”[MeSH Terms] OR serology[Title/Abstract] OR serological[Title/Abstract] OR “antibodies, viral”[MeSH Terms] OR antibody[Title/Abstract] OR antibodies[Title/Abstract]) AND ((“swine”[MeSH Terms] OR pigs[Title/Abstract]) OR (“sus scrofa”[MeSH Terms] OR (wild[Title/Abstract] AND (boars[Title/Abstract] OR swine[Title/Abstract]))))	1372
Scopus	(TITLE-ABS (“porcine reproductive and respiratory syndrome virus”) OR TITLE-ABS (PRRSV) OR TITLE-ABS (“porcine reproductive and respiratory syndrome”)) AND (TITLE-ABS (serolog*) OR TITLE-ABS (antibody) OR TITLE-ABS (antibodies) OR TITLE-ABS (serum)) AND (TITLE-ABS (swine) OR TITLE-ABS (pigs) OR (TITLE-ABS (wild) AND (TITLE-ABS (boar*) OR TITLE-ABS (pig*) OR TITLE-ABS (“Sus scrofa”))))	1518
Web of Science	TS = (“porcine reproductive and respiratory syndrome virus” OR PRRSV OR “porcine reproductive and respiratory syndrome”) AND TS = (serology OR serological OR antibody OR antibodies OR serum) AND TS = (“wild boar” OR “Sus scrofa” OR swine OR pigs) AND TS = (detection OR prevalence)	513

## Data Availability

The original contributions included in the metanalysis are reported in the references [39,40,41,42,44,45,46,47,48,49,50,51,52,53,54,55,56,57,58,59,60,61,62,63,64,65,66,67,68,69,70,71,72,73,74,75,76,77,78,79,80,81,82,83,84,85,86,87,88,89,90,91,92,93,94,95,96,97,98,99,100,101,102,103,104,105,106,107,108,109,110,111,112,113,114,115,116,117,118,119,120] and in the Supplementary Materials. Further inquiries can be directed to the corresponding authors.

## Supplementary Materials

The following supporting information can be downloaded at: https://www.mdpi.com/article/10.3390/vetsci13030304/s1, Supplementary Material S1: Systematic review and Meta-Analysis Protocol (PRISMA-P); Supplementary Material S2: Details of the eligible studies on the serological prevalence of PRRSV in swine and wild boars, sorted by continent; Supplementary Material S3: Outliers identification for seroprevalence studies on PRRSV in suids; Supplementary Material S4: Sub-group analyses of eligible PRRSV serological studies.
