# Supplementary material for: A Systematic Review and Meta-Analysis on the Global Seroprevalence of Porcine Reproductive and Respiratory Virus (PRRSV) in Pigs and Wild Boars: A Widespread and Impactful Swine Virus
Source: Vet Sci. 2026 Mar 23;13(3):304. doi: 10.3390/vetsci13030304 (PMC13030171; doi:10.3390/vetsci13030304)
Supplement: Supplementary file 1 [file vetsci-13-00304-s001.zip › Supplementary Material 2.pdf]

Supplementary Material S2: Details of the eligible studies on the serological prevalence of PRRSV in swine and wild boars, sorted by continent. References are provided in the back matter section.

First Author	Year (sampling)	Subspecies	Country (Continent)	Sample size	Number of positive	Prevalence	Method
**AFRICA**							
Aiki-Raji et al. (2018)	2014-2015	Pig	Nigeria	368	198	53.8	ELISA (C)
Dione et al. (2018)	2015	Pig	Uganda (E+I)	522	8	1.53	ELISA (C)
Oba et al. (2023)	2018	Pig	Uganda	259	36	13.9	ELISA (C-1-2)
**AMERICAS**							
Baroch et al. (2015)	2011-2012	Wild Boar	USA (E)	162	4	2.47	ELISA (C-1-2)
Campbell et al. (2008)	2006-2007	Wild Boar	USA (E)	409	3	0.73	ELISA (C)
Cho et al. (1993)	1993	Pig	USA	2787	979	35.13	IFAT (H)
Ciacci-Zanella et al. (2004)	1990-2000	Pig	Brazil	3785	27	0.71	ELISA (C-1-2)
Corn et al. (2009)	2007	Wild Boar	USA (E)	120	1	0.83	ELISA (C)
Cruz (2006)	2006	Pig	Colombia	1658	71	4.28	ELISA (C)
Gava et al. (2022)	2008-2020	Pig	Brazil (E+I)	12841	15	0.12	ELISA (C-1-2)
Kwiecien et al. (2017)	2013-2015	Pig	Venezuela	1287	464	36.05	ELISA (C-1-2)
McGregor et al. (2015)	2014	Wild Boar	Canada (E)	44	0	0	ELISA (C-1-2)

Martínez-Bautista et al. (2018)	2017	Pig	Mexico	3420	1477	43.19	ELISA (H)
Mejía Silva et al. (2012)	n.a.	Pig	Venezuela	393	10	2.54	ELISA (C-1-2)
Meléndez et al. (2008)	2002	Pig	Mexico	300	83	27.67	ELISA (C-1-2)
Meléndez et al. (2021)	n.a.	Pig	Costa Rica	596	344	57.72	ELISA (C-1-2)
Pedersen et al. (2018)	2013-2015	Wild Board	USA	55066	68	1.24	ELISA (C-1-2)
Pérez-Rivera et al. (2017)	n.a.	Wild Boar	Mexico	70	0	0	ELISA (C-2)
Quevedo et al. (2018)	2016	Pig	Perù	4526	783	17.3	ELISA (C)
Saliki et al. (1998)	1996	Wild Boar	USA (E)	117	2	1.71	ELISA (C-1-2)
Sookhoo et al. (2017)	2013-2015	Pig	Trinidad and Tobago	309	0	0	ELISA (C-2)
Wayne et al. (2012)	2004	Pig	USA	661	324	49.02	ELISA (H)
Wyckoff et al. (2009)	2004-2006	Wild Boar	USA (E)	77	1	1.3	ELISA (H)
**ASIA**							
Albayrak et al. (2013)	2012	Wild Boar	Turkey (E)	93	2	2.15	ELISA (C-1-2)
Cheon et al. (1997)	1996	Pig	Republic of Korea	2132	964	45.22	ELISA (C)
Choi et al. (2012)	2011	Wild Boar	Republic of Korea (E)	267	4	1.5	ELISA (C-1-2)
Chumsang et al. (2021)	2016-2017	Pig	Thailand (E)	237	32	13.5	ELISA (C-1-2)
Han et al. (2021)	2019	Pig	China	1206	1065	88.31	ELISA (C-1-2)

Ho et al. (1999)	1995-1998	Pig	China	180	18	10	IFAT (H)
Jasbir et al. (1995)	1994-1995	Pig	Malaysia	200	97	48.5	ELISA (C)
Kamakawa et al. (2006)	1999-2002	Pig	Vietnam (E+I)	478	37	7.74	ELISA (C-1-2)
Kaneko et al. (2022)	2020	Wild Boar	Japan (E)	140	0	0	ELISA (C-1-2)
Kaur et al. (2019)	n.a.[§]	Pig	India	90	20	22.22	ELISA (C-1-2)
Kim et al. (2002)	2001	Pig	Republic of Korea	3391	1765	52.05	IFAT (H)
Kitamura et al. (2022)	2010-2021	Wild Boar	Japan (E)	453	3	0.66	ELISA (C-1-2)
Kukushkin et al. (2008)	2002-2005	Wild Boar	Russia (E)	90	0	0	ELISA (C-1-2)
Lee et al. (2024)	2021-2024	Pig	Republic of Korea	57	46	80.7	ELISA (C-1-2)
Lalhruaipuii et al. (2020)	2018-2019	Pig	India	420	117	27.86	ELISA (C-1-2)
Lee et al. (2020)	2020	Pig	Vietnam	600	135	22.5	ELISA (C-1-2)
Mahesh et al. (2015)	2014	Pig	Nepal (E+I)	200	37	18.5	LF-ICA (C-1-2)
Monger et al. (2014)	2011-2012	Pig	Bhutan (E+I)	465	0	0	IPMA (H)
Mukherjee et al. (2018)	2014-2015	Pig	India	1898	62	3.27	ELISA (C)
Prajapati et al. (2023)	2020-2021	Pig	Nepal	180	37	20.56	ELISA (C-1-2)
Tummaruk and Tantilertcharoen (2012)	2004-2017	Pig	Thailand	5664	4492	79.31	ELISA (C-1-2)
Sharma et al. (2016)	2011	Pig	Nepal (E+I)	98	31	31.63	ELISA (C-1-2)
Siengsanan-Lamont et al. (2022)	2019-2020	Pig	Cambodia	655	532	81.22	ELISA (C-1-2)

Wiratsudakul et al. (2013)	2009	Wild Boar	Thailand (E+I)	52	13	25	ELISA (C)
Yu et al. (2022)	n.a.	Pig	China (I)	627	300	47.85	ELISA (C-1-2)
Zhao et al. (2022)	2017-2021	Pig	China	14134	8741	61.84	ELISA (C)
**OCEANIA**							
Garner et al. (1997)	1996	Pig	Australia	875	4	0.46	ELISA (C)
**EUROPE**							
Albina et al. (2000)	1993-1995	Wild Boar	France (E+I)	303	25	8.25	ELISA (H)
Bálint et al. (2024)	2011-2023	Wild Boar	Hungary (E)	2842	12	0.42	ELISA (C-1-2)
Boadella et al. (2012)	2000-2009	Wild Boar	Spain (E)	407	9	2.21	ELISA (C-1-2)
Buitkuvienė et al. (2014)	2008-2011	Pig	Lithuan (I)	8704	373	4.29	ELISA (C-1-2)
	2008-2011	Wild Boar	Lithuan (E)	1022	65	6.36	ELISA (C-1-2)
Cano-Manuel et al. (2014)	2002-2008	Wild Boar	Spain (I)	1100	21	1.91	ELISA (C-1-2)
Carlsson et al. (2009)	2007	Pig	Sweden	16739	0	0	ELISA (C-1-2)
Closa-Sebastià et al. (2011)	2004-2007	Wild Boar	Spain (E)	265	8	3.02	ELISA (C-1-2)
Corbellini et al. (2006)	2001-2004	Pig	Switzerland	48622	17	0.03	ELISA (C-1-2)
Correia-Gomes et al. (2022)	2006-2013	Pig	Scotland	2811	1071	38.1	ELISA (C-1-2)
Duinhof et al. (2011)	2005	Pig	The Netherlands	1002	578	57.68	ELISA (C-1-2)
Evans et al. (2008)	2003-2004	Pig	United Kingdom	4852	1958	40.35	ELISA (C-1)

Fabisiak et al. (2013)	2006-2007	Wild Boar	Poland	142	1	0.7	ELISA (C)
Ferrara et al. (2023)	2022	Pig	Italy (E+I)	438	73	16.67	ELISA (C-1-2)
Foti et al. (2008)	2004	Pig	Italy (E+I)	912	289	31.69	ELISA (C)
Groschup et al. (1993)	1991-1992	Pig	Germany	265	183	69.06	IPMA (H)
Gutiérrez-Martín et al. (2000)	1998	Pig	Spain	198	175	88.38	ELISA (C-1)
Hälli et al. (2012)	2005-2008	Wild Boar	Finland (E)	303	0	0	ELISA (C-1-2)
Hammer et al. (2012)	2008-2009	Wild Boar	Germany (E)	203	1	0.49	ELISA (C-1-2)
Kaden et al. (2009)	1997-2005	Wild Boar	Germany (E)	2222	42	1.89	ELISA (C-1-2)
Lopes Antunes et al. (2015)	2007-2010	Pig	Denmark	391723	174460	44.53	ELISA (C-1-2)
Marinou et al. (2015)	2013	Wild Boar	Greece (I)	321	18	5.61	ELISA (C-1-2)
Meemken et al. (2014)	2010-2011	Pig	Germany	688	84	12.21	ELISA (C-1-2)
Montagnaro et al. (2010)	2005-2006	Wild Boar	Italy (E)	342	129	37.72	ELISA (C-1-2)
Nemes et al. (2019)	2012-2015	Pig	Hungary (E)	115257	3943	3.42	ELISA (C-1-2)
Powell et al. (2016)	2013	Pig	United Kingdom	621	362	58.29	ELISA (C-1-2)
Rodríguez-Prieto et al. (2013)	2002-2010	Wild Boar	Spain (E)	294	7	2.38	ELISA (C-1-2)
	2002-2010	Pig	Spain (I)	80	1	1.25	ELISA (C-1-2)
Roic et al. (2012)	2005-2010	Wild Boar	Croatia (E)	753	35	4.65	ELISA (C-1-2)
Ruiz-Fons et al. (2006)	2000-2003	Wild Boar	Spain (E+I)	123	0	0	ELISA (C-1-2)

Sattler et al. (2012)	n.a.	Wild Boar	Germany	94	1	1.06	ELISA (C-1-2)
Stankevicius et al. (2014)	2009-2013	Pig	Lithuan (I)	9856	426	4.32	ELISA (C-1-2)
	2009-2013	Wild Boar	Lithuan (E)	1357	73	5.38	ELISA (C-1-2)
Štukelj et al. (2014)	2010-2011	Wild Boar	Slovenia (E)	184	0	0	ELISA (C-1-2)
Touloudi et al. (2015)	2006-2010	Wild Boar	Greece (E)	94	12	12.77	ELISA (C-1-2)
Vengust et al. (2006)	2003-2004	Wild Boar	Slovenia (E)	178	0	0	ELISA (C-1-2)
Vicente et al. (2002)	1999-2000	Wild Boar	Spain (E)	78	0	0	ELISA (C-1-2)
Wu et al. (2011)	2008-2010	Wild Boar	Switzerland (E)	233	1	0.43	ELISA (C-1-2)
Zupancić et al. (2002)	1999	Wild Boar	Croatia (E)	44	0	0	ELISA (C-1-2)

[§] n.a.: not available; E=extensive/wild/free-ranging; I=intensive; H= in-house assay; C= commercial assay; 1=ability to detect antibodies against European strains; 2=ability to detect antibodies against American strains

References

Aiki-Raji, C, Adebiyi, A, Abiola, O, Oluwayelu, D (2018) Prevalence of Porcine Reproductive and Respiratory Syndrome Virus and Porcine Parvovirus Antibodies in Commercial Pigs, Southwest Nigeria. *Beni-Suef University Journal of Basic and Applied Sciences, 7*. doi:10.1016/j.bjbas.2017.07.006

Albayrak, H, Ozan, E, Cavunt, A (2013) A Serological Survey of Selected Pathogens in Wild Boar (Sus Scrofa) in Northern Turkey. *European Journal of Wildlife Research, 59*(6), 893-897. doi:10.1007/s10344-013-0743-6

Albina, E, Mesplède, A, Chenut, G, Le Potier, MF, Bourbao, G, Le Gal, S, Leforban, Y (2000) A Serological Survey on Classical Swine Fever (Csf), Aujeszky's Disease (Ad) and Porcine Reproductive and Respiratory Syndrome (Prrs) Virus Infections in French Wild Boars from 1991 to 1998. *Veterinary Microbiology, 77*(1), 43-57. doi:10.1016/S0378-1135(00)00255-8

Bálint, Á, Csányi, S, Nemes, I, Bijl, H, Szabó, I (2024) Investigation of Prrs Virus Infection in Hungarian Wild Boar Populations During Its Eradication from Domestic Pig Herds. *Animals, 14*(11), 1537. doi:10.3390/ani14111537

Baroch, JA, Gagnon, CA, Lacouture, S, Gottschalk, M (2015) Exposure of Feral Swine (Sus Scrofa) in the United States to Selected Pathogens. *Canadian Journal of Veterinary Research, 79*(1), 74-78.

Boadella, M, Ruiz-Fons, JF, Vicente, J, Martín, M, Segalés, J, Gortazar, C (2012) Seroprevalence Evolution of Selected Pathogens in Iberian Wild Boar. *Transboundary and Emerging Diseases, 59*(5), 395-404. doi:10.1111/j.1865-1682.2011.01285.x

Buitkuvienė, J, Deltuvytienė, J, Čepulienė, R, Žilionytė, V, Mozūraitytė, J, Pridotkas, G, Stankevičius, A (2014) Serological Survey on Porcine Reproductive and Respiratory Syndrome Virus (Prrsv) in Lithuanian Pigs and Wild Boars. *Veterinarija ir Zootechnika, 67*(89).

Campbell, TA, DeYoung, RW, Wehland, EM (2008) Feral Swine Exposure to Selected Viral and Bacterial Pathogens in Southern Texas. *JSHAP, 16*(6), 312-315.

Cano-Manuel, FJ, López-Olvera, J, Fandos, P, Soriguer, RC, Pérez, JM, Granados, JE (2014) Long-Term Monitoring of 10 Selected Pathogens in Wild Boar (Sus Scrofa) in Sierra Nevada National Park, Southern Spain. *Veterinary Microbiology, 174*(1), 148-154. doi:10.1016/j.vetmic.2014.06.017

Carlsson, U, Wallgren, P, Renström, LHM, Lindberg, A, Eriksson, H, Thorén, P, Eliasson-Selling, L, Lundeheim, N, Nörregard, E, Thörn, C, Elvander, M (2009) Emergence of Porcine Reproductive and Respiratory Syndrome in Sweden: Detection, Response and Eradication. *Transboundary and Emerging Diseases, 56*(4), 121-131. doi:10.1111/j.1865-1682.2008.01065.x

Cheon, D-S, Chae, C, Lee, Y-S (1997) Seroprevalence of Antibody to Porcine Reproductive and Respiratory Syndrome Virus Using Enzyme-Linked Immunosorbent Assay in Selected Herds in Korea. *Journal of Veterinary Diagnostic Investigation, 9*(4), 434-436. doi:10.1177/104063879700900419

Cho, SH, Freese, WR, Yoon, IJ, Trigo, AV, Joo, HS (1993) Seroprevalence of Indirect Fluorescent Antibody to Porcine Reproductive and Respiratory Syndrome Virus in Selected Swine Herds. *Journal of Veterinary Diagnostic Investigation, 5*(2), 259-260. doi:10.1177/104063879300500220

Choi, E-J, Lee, C-H, Hyun, B-H, Kim, J-J, Lim, S-I, Song, J-Y, Shin, Y-K (2012) A Survey of Porcine Reproductive and Respiratory Syndrome among Wild Boar Populations in Korea. *Journal of Veterinary Science, 13*(4), 377-383. doi:10.4142/jvs.2012.13.4.377

Chumsang, S, Na Lampang, K, Srikitjakarn, L, Pringproa, K (2021) Seroprevalence of the Viral Pig Diseases among Backyard Pigs in Chiang Mai, Thailand. *Preventive Veterinary Medicine, 190*, 105330. doi:10.1016/j.prevetmed.2021.105330

Ciacci-Zanella, JR, Trombetta, C, Vargas, I, Mariano da Costa, DE (2004) Lack of Evidence of Porcine Reproductive and Respiratory Syndrome Virus (Prrsv) Infection in Domestic Swine in Brazil. *Ciência Rural, 32*(2), 449–455.

Closa-Sebastià, F, Casas-Díaz, E, Cuenca, R, Lavín, S, Mentaberre, G, Marco, I (2011) Antibodies to Selected Pathogens in Wild Boar (Sus Scrofa) from Catalonia (Ne Spain). *European Journal of Wildlife Research, 57*(4), 977-981. doi:10.1007/s10344-010-0491-9

Corbellini, LG, Schwermer, H, Presi, P, Thür, B, Stärk, KDC, Reist, M (2006) Analysis of National Serological Surveys for the Documentation of Freedom from Porcine Reproductive and Respiratory Syndrome in Switzerland. *Veterinary Microbiology, 118*(3), 267-273. doi:10.1016/j.vetmic.2006.07.018

Corn, JL, Cumbee, JC, Barfoot, R, Erickson, GA (2009) Pathogen Exposure in Feral Swine Populations Geographically Associated with High Densities of Transitional Swine Premises and Commercial Swine Production. *Journal of Wildlife Diseases, 45*(3), 713-721. doi:10.7589/0090-3558-45.3.713

Correia-Gomes, C, Duncan, A, Ward, A, Pearce, M, Eppink, L, Webster, G, McGowan, A, Thomson, J (2022) Porcine Reproductive and Respiratory Syndrome Virus Seroprevalence in Scottish Finishing Pigs between 2006 and 2018. *Veterinary Record, 190*(7), e349. doi:10.1002/vetr.349

Cruz, MC (2006) Prevalencia Serológica Del Síndrome Reproductivo Y Respiratorio Porcino (Prrs) En Cerdos De Explotaciones Extensivas De Colombia. *Rev Med Vet Zoot., 53*, 33-41.

Dione, M, Masembe, C, Akol, J, Amia, W, Kungu, J, Lee, HS, Wieland, B (2018) The Importance of on-Farm Biosecurity: Sero-Prevalence and Risk Factors of Bacterial and Viral Pathogens in Smallholder Pig Systems in Uganda. *Acta Tropica, 187*, 214-221. doi:10.1016/j.actatropica.2018.06.025

Duinhof, TF, van Schaik, G, van Esch, EJB, Wellenberg, GJ (2011) Detection of Prrsv Circulation in Herds without Clinical Signs of Prrs: Comparison of Five Age Groups to Assess the Preferred Age Group and Sample Size. *Veterinary Microbiology, 150*(1), 180-184. doi:10.1016/j.vetmic.2011.01.001

Evans, CM, Medley, GF, Green, LE (2008) Porcine Reproductive and Respiratory Syndrome Virus (Prrsv) in Gb Pig Herds: Farm Characteristics Associated with Heterogeneity in Seroprevalence. *BMC Veterinary Research, 4*(1), 48. doi:10.1186/1746-6148-4-48

Fabisiak, M, Podgórska, K, Skrzypiec, E, Szczotka, A, Stadejek, T (2013) Detection of Porcine Circovirus Type 2 (Pcv2) and Porcine Reproductive and Respiratory Syndrome Virus (Prrsv) Antibodies in Meat Juice Samples from Polish Wild Boar (Sus Scrofa L.). *Acta Veterinaria Hungarica, 61*(4), 529-536. doi:10.1556/avet.2013.027

Ferrara, G, D'Anza, E, Rossi, A, Improda, E, Iovane, V, Pagnini, U, Iovane, G, Montagnaro, S (2023) A Serological Investigation of Porcine Reproductive and Respiratory Syndrome and Three Coronaviruses in the Campania Region, Southern Italy. *Viruses, 15*(2), 300.

Foti, M, Bottari, T, Daidone, A, Rinaldo, D, De Leo, F, Foti, S, Giacopello, C (2008) Serological Survey on Aujeszky's Disease, Swine Influenza and Porcine Reproductive and Respiratory Syndrome Virus Infections in Italian Pigs. *Polish Journal of Veterinary Sciences, 11*(4), 323-325.

Garner, MG, Gleeson, LJ, Holyoake, PK, Cannon, RM, Doughty, WJ (1997) A National Serological Survey to Verify Australia's Freedom from Porcine Reproductive and Respiratory Syndrome. *Australian Veterinary Journal, 75*(8), 596-600. doi:10.1111/j.1751-0813.1997.tb14202.x

Gava, D, Caron, L, Schaefer, R, Silva, VS, Weiblen, R, Flores, EF, de Lima, M, Takeda, GZ, Ciacci-Zanella, JR (2022) A Retrospective Study of Porcine Reproductive and Respiratory Syndrome Virus Infection in Brazilian Pigs from 2008 to 2020. *Transboundary and Emerging Diseases, 69*(2), 903-907. doi:10.1111/tbed.14036

Groschup, MH, Brun, A, Haas, B (1993) Serological Studies on the Potential Synergism of Porcine Reproductive and Respiratory Syndrome Virus and Influenza-, Corona- and Paramyxoviruses in the Induction of Respiratory Symptoms in Swine. *Journal of Veterinary Medicine, Series B, 40*(1-10), 681-689. doi:10.1111/j.1439-0450.1993.tb00192.x

Gutiérrez-Martín, CB, RodrÍguez-Delgado, Ó, ÁLvarez-Nistal, D, De La Puente-Redondo, VA, GarcÍa-Rioja, F, MartÍn-Vicente, J, RodrÍguez Ferri, EF (2000) Simultaneous Serological Evidence of Actinobacillus Pleuropneumoniae, Prrs, Aujeszky's Disease and Influenza Viruses in Spanish Finishing Pigs. *Research in Veterinary Science, 68*(1), 9-13. doi:10.1053/rvsc.1999.0326

Hälli, O, Ala-Kurikka, E, Nokireki, T, Skrzypczak, T, Raunio-Saarnisto, M, Peltoniemi, OA, Heinonen, M (2012) Prevalence of and Risk Factors Associated with Viral and Bacterial Pathogens in Farmed European Wild Boar. *The Veterinary Journal, 194*(1), 98-101. doi:10.1016/j.tvjl.2012.03.008

Hammer, R, Ritzmann, M, Palzer, A, Lang, C, Hammer, B, Pesch, S, Ladinig, A (2012) Porcine Reproductive and Respiratory Syndrome Virus and Porcine Circovirus Type 2 Infections in Wild Boar (Sus Scrofa) in Southwestern Germany. *Journal of Wildlife Diseases, 48*(1), 87-94. doi:10.7589/0090-3558-48.1.87

Han, D, Yang, H, Yang, Y, Ye, L, Dong, J, Zhang, C, Zhu, H, Yin, S, Dong, X, Su, F, Xin, J, Ai, J (2021) Porcine Reproductive and Respiratory Syndrome Virus (Prrsv) Antibody Levels in Large Swine Farms in Selected Regions of Yunnan Province, China. *Medycyna Weterynaryjna, 77*, 497-501. doi:10.21521/mw.6581

Ho, S-h, Jun, Y-c, Park, C-k, Lee, C-h, Bae, J-h (1999) Prevalence of Tissue Antigen and Serum Antibody for Porcine Reproductive and Respiratory Syndrome in Cheju. *Korean Journal of Veterinary Research, 39*(4), 760-764.

Jasbir, S, Hussin, AA, Arunasalam, V (1995) Seroprevalence of Porcine Reproductive and Respiratory Syndrome (Prrs). *Malaysian Journal of Veterinary Research, 7*, 81-82.

Kaden, V, Lange, E, Hänel, A, Hlinak, A, Mewes, L, Hergarten, G, Irsch, B, Dedek, J, Bruer, W (2009) Retrospective Serological Survey on Selected Viral Pathogens in Wild Boar Populations in Germany. *European Journal of Wildlife Research, 55*(2), 153-159. doi:10.1007/s10344-008-0229-0

Kamakawa, A, Ho, TV, Yamada, S (2006) Epidemiological Survey of Viral Diseases of Pigs in the Mekong Delta of Vietnam between 1999 and 2003. *Veterinary Microbiology, 118*(1-2), 47-56. doi:10.1016/j.vetmic.2006.07.003

Kaneko, F, Kitamura, N, Suzuki, K, Kato, M (2022) Serological Survey of Antibodies to Four Pathogens in Wild Boars in Nagano Prefecture, Japan. *Journal of Veterinary Medical Science, 84*(6), 855-859. doi:10.1292/jvms.22-0035

Kaur, A, Mahajan, V, Leishangthem, G, Singh, N, Banga, H, Filia, G (2019) Seroprevalence Study for Detection of Porcine Reproductive and Respiratory Syndrome Virus Antibodies in Pig Population of Punjab. *Haryana Veterinarian, 58*(1), 122–123. doi:10.14202/vetworld.2016.827-831

Kim, SM, Han, TU, Kang, SY, Shin, KS, Kim, CJ, Kim, JT, Kim, HS (2002) Seroprevalence of Antibody to Procine Reproductive and Respiratory Syndrome Virus in Diagnostic Submissions. *Journal of Veterinary Science, 3*(3), 159-161.

Kitamura, Y, Saito, T, Tanaka, E, Takashima, Y (2022) A Serological Survey of Porcine Reproductive and Respiratory Syndrome Virus in Wild Boar in Gifu Prefecture, Japan. *Journal of Veterinary Medical Science, 84*(10), 1406-1409. doi:10.1292/jvms.21-0554

Kukushkin, S, Kanshina, A, Timina, A, Baybikov, T, Mikhalishin, V (2008) Investigation of Wild Boar (Sus Scrofa) for Porcine Reproductive and Respiratory Syndrome in Some Territories of Russia. *European Journal of Wildlife Research, 54*(3), 515-518. doi:10.1007/s10344-007-0159-2

Kwiecien, EJ, Mejía-Silva, W, Quintero-Moreno, A, Gutierrez, C (2017) Estudio De La Respuesta Serológica Contra El Virus Del Síndrome Reproductivo Y Respiratorio Porcino (Prrs) En Cerdos Bajo Condiciones Tropicales. *Revista Científica, 27*(5), 282-293.

Lalhruaipuii, K, Shakuntala, I, Sen, A (2020) Seroprevalence of Porcine Reproductive and Respiratory Syndrome Virus and Classical Swine Fever Virus in Pigs of Mizoram, India. *Journal of Environmental Biology, 41*, 915-920. doi:10.22438/jeb/4(SI)/MS_1920

Lee, HS, Bui, VN, Nguyen, HX, Bui, AN, Hoang, TD, Nguyen-Viet, H, Grace Randolph, D, Wieland, B (2020) Seroprevalences of Multi-Pathogen and Description of Farm Movement in Pigs in Two Provinces in Vietnam. *BMC Veterinary Research, 16*(1), 15. doi:10.1186/s12917-020-2236-7

Lee, YB, Kim, JW, Jo, W, Kang, TK, Sung, M, Kim, K, Park, NH, Lee, GH (2024) Assessment of Prrsv and Pcv2 Seroprevalence and Antigen Prevalence in Minipigs at Laboratory-Animal Production Facilities. *Journal of Advanced Veterinary and Animal Research, 11*(4), 1017-1022. doi:10.5455/javar.2024.k852

Lopes Antunes, AC, Halasa, T, Lauritsen, KT, Kristensen, CS, Larsen, LE, Toft, N (2015) Spatial Analysis and Temporal Trends of Porcine Reproductive and Respiratory Syndrome in Denmark from 2007 to 2010 Based on Laboratory Submission Data. *BMC Veterinary Research, 11*(1), 303. doi:10.1186/s12917-015-0617-0

Mahesh, K, Bhoj, J, Swoyam, S, Meera, P, Dipak, K, Santosh, D (2015) Sero-Prevalence of Porcine Reproductive and Respiratory Syndrome (Prrs) in Pigs of Different Developmental Regions of Nepal. *International journal of applied sciences and biotechnology, 3*, 218-222. doi:10.3126/ijasbt.v3i2.12539

Marinou, KA, Papatsiros, VG, Gkotsopoulos, EK, Odatzoglou, PK, Athanasiou, LV (2015) Exposure of Extensively Farmed Wild Boars (Sus Scrofa Scrofa) to Selected Pig Pathogens in Greece. *Veterinary Quarterly, 35*(2), 97-101. doi:10.1080/01652176.2015.1022666

Martínez-Bautista, NR, Sciutto-Conde, E, Cervantes-Torres, J, Segura-Velázquez, R, Mercado García, MC, Ramírez-Mendoza, H, Trujillo Ortega, ME, Delgadillo Alvarez, J, Castillo-Juárez, H, Sanchez-Betancourt, JI (2018) Phylogenetic Analysis of Orf5 and Orf7 of Porcine Reproductive and Respiratory Syndrome (Prrs) Virus and the Frequency of Wild-Type Prrs Virus in México. *Transboundary and Emerging Diseases, 65*(4), 993-1008. doi:10.1111/tbed.12831

McGregor, GF, Gottschalk, M, Godson, DL, Wilkins, W, Bollinger, TK (2015) Disease Risks Associated with Free-Ranging Wild Boar in Saskatchewan. *Canadian Veterinary Journal, 56*(8), 839-844.

Meemken, D, Tangemann, AH, Meermeier, D, Gundlach, S, Mischok, D, Greiner, M, Klein, G, Blaha, T (2014) Establishment of Serological Herd Profiles for Zoonoses and Production Diseases in Pigs by “Meat Juice Multi-Serology”. *Preventive Veterinary Medicine, 113*(4), 589-598. doi:10.1016/j.prevetmed.2013.12.006

Mejía Silva, W, Calatayud, D, Zapata, D, Quintero Moreno, A, Torres, P, Chango, M (2012) Seroprevalencia De La Enfermedad De Aujeszky Y Del Síndrome Respiratorio Y Reproductivo Porcino (Prrs) En Granjas Porcinas Del Municipio Mauroa Del Estado Falcón. *Revista Científica, 22*, 139-144.

Meléndez, JAS, Arias, JL, Andrade, HF, Ramírez, RÁ (2008) Presencia De Animales Seropositivos Al Síndrome Reproductivo Y Respiratorio Porcino En Nuevo León. *Veterinaria México, 39*(2), 215-221.

Meléndez, R, Guzmán, M, Jiménez, C, Piche, M, Jiménez, E, León, B, Cordero, JM, Ramirez-Carvajal, L, Uribe, A, Van Nes, A, Stegeman, A, Vernooij, H, Romero-Zúñiga, JJ (2021) Seroprevalence of Porcine Reproductive and Respiratory Syndrome Virus on Swine Farms in a Tropical Country of the Middle Americas: The Case of Costa Rica. *Tropical Animal Health and Production, 53*(4), 441. doi:10.1007/s11250-021-02799-9

Monger, VR, Stegeman, JA, Koop, G, Dukpa, K, Tenzin, T, Loeffen, WLA (2014) Seroprevalence and Associated Risk Factors of Important Pig Viral Diseases in Bhutan. *Preventive Veterinary Medicine, 117*(1), 222-232. doi:10.1016/j.prevetmed.2014.07.005

Montagnaro, S, Sasso, S, De Martino, L, Longo, M, Iovane, V, Ghiurmino, G, Pisanelli, G, Nava, D, Baldi, L, Pagnini, U (2010) Prevalence of Antibodies to Selected Viral and Bacterial Pathogens in Wild Boar (Sus Scrofa) in Campania Region, Italy. *Journal of Wildlife Diseases, 46*(1), 316-319. doi:10.7589/0090-3558-46.1.316

Mukherjee, P, Karam, A, Singh, U, Chakraborty, AK, Huidrom, S, Sen, A, Sharma, I (2018) Seroprevalence of Selected Viral Pathogens in Pigs Reared in Organized Farms of Meghalaya from 2014 to 16. *Veterinary World, 11*(1), 42-47. doi:10.14202/vetworld.2018.42-47

Nemes, I, Molnár, T, Abonyi, T, Terjék, Z, Bálint, Á, Szabó, I (2019) Eradication of Prrs from Backyard Swine Herds in Hungary between 2012 and 2018. *Acta Veterinaria Hungarica, 67*(4), 543-552. doi:10.1556/004.2019.053

Oba, P, Wieland, B, Mwiine, FN, Erume, J, Dione, MM (2023) Co-Infections of Respiratory Pathogens and Gastrointestinal Parasites in Smallholder Pig Production Systems in Uganda. *Parasitology Research, 122*(4), 953-962. doi:10.1007/s00436-023-07797-4

Pedersen, K, Miller, RS, Musante, AR (2018) Antibody Evidence of Porcine Reproductive and Respiratory Syndrome Virus Detected in Sera Collected from Feral Swine (Sus Scrofa) across the United States. *JSHAP, 26*(1), 41-44.

Pérez-Rivera, CM, López, MS, Arnaud-Franco, G, Carreón-Nápoles, R (2017) Detection of Antibodies against Pathogens in Feral and Domestic Pigs (Sus Scrofa) at the Sierra La Laguna Biosphere Reserve, Mexico. *Veterinaria México, 4*(1). doi:10.22201/fmvz.24486760e.2017.378

Powell, LF, Cheney, TEA, Williamson, S, Guy, E, Smith, RP, Davies, RH (2016) A Prevalence Study of Salmonella Spp., Yersinia Spp., Toxoplasma Gondii and Porcine Reproductive and Respiratory Syndrome Virus in Uk Pigs at Slaughter. *Epidemiology and Infection, 144*(7), 1538-1549. doi:10.1017/S0950268815002794

Prajapati, M, Acharya, MP, Yadav, P, Frossard, J-P (2023) Farm Characteristics and Sero-Prevalence of Porcine Reproductive and Respiratory Syndrome Virus (Prrsv) Antibodies in Pigs of Nepal. *Veterinary Medicine and Science, 9*(1), 174-180. doi:10.1002/vms3.1011

Quevedo, V, M., Mantilla, S, J., Portilla, J, K., Villacaqui, A, R., Rivera, G, H. (2018) Seroprevalencia Del Virus Del Síndrome Reproductivo Y Respiratorio Porcino En Cerdos De Crianza No Tecnificada Del Perú. *Revista De Investigaciones Veterinarias Del Perú, 29*(2), 643-651.

Rodríguez-Prieto, V, Kukielka, D, Martínez-López, B, de las Heras, AI, Barasona, JÁ, Gortázar, C, Sánchez-Vizcaíno, JM, Vicente, J (2013) Porcine Reproductive and Respiratory Syndrome (Prrs) Virus in Wild Boar and Iberian Pigs in South-Central Spain. *European Journal of Wildlife Research, 59*(6), 859-867. doi:10.1007/s10344-013-0739-2

Roic, B, Jemersic, L, Terzic, S, Keros, T, Balatinec, J, Florijancic, T (2012) Prevalence of Antibodies to Selected Viral Pathogens in Wild Boars (Sus Scrofa) in Croatia in 2005–06 and 2009–10. *Journal of Wildlife Diseases, 48*(1), 131-137. doi:10.7589/0090-3558-48.1.131

Ruiz-Fons, F, Vicente, J, Vidal, D, Höfle, U, Villanúa, D, Gauss, C, Segalés, J, Almería, S, Montoro, V, Gortázar, C (2006) Seroprevalence of Six Reproductive Pathogens in European Wild Boar (Sus Scrofa) from Spain: The Effect on Wild Boar Female Reproductive Performance. *Theriogenology, 65*(4), 731-743. doi:10.1016/j.theriogenology.2005.07.001

Saliki, JT, Rodgers, SJ, Eskew, G (1998) Serosurvey of Selected Viral and Bacterial Diseases in Wild Swine from Oklahoma. *Journal of Wildlife Diseases, 34*(4), 834-838. doi:10.7589/0090-3558-34.4.834

Sattler, T, Sailer, E, Wodak, E, Schmoll, F (2012) Serological Detection of Emerging Viral Infections in Wild Boars from Different Hunting Regions of Southern Germany. *Tierarztl Prax Ausg G Grosstiere Nutztiere, 40*(1), 27-32.

Sharma, BK, Manandhar, S, Devleesschauwer, B (2016) Serological Evidence of Type 2 (North American Genotype) Porcine Reproductive and Respiratory Syndrome Virus in Nepal. *Tropical Animal Health and Production, 48*(3), 663-666. doi:10.1007/s11250-015-0986-1

Siengsanan-Lamont, J, Tum, S, Kong, L, Selleck, PW, Gleeson, LJ, Blacksell, SD (2022) Abattoir-Based Serological Surveillance for Transboundary and Zoonotic Diseases in Cattle and Swine in Cambodia: A Pilot Study in Phnom Penh Province During 2019 and 2020. *Tropical Animal Health and Production, 54*(5), 316. doi:10.1007/s11250-022-03309-1

Sookhoo, JRV, Brown-Jordan, A, Blake, L, Holder, RB, Brookes, SM, Essen, S, Carrington, CVF, Brown, IH, Oura, CAL (2017) Seroprevalence of Economically Important Viral Pathogens in Swine Populations of Trinidad and Tobago, West Indies. *Tropical Animal Health and Production, 49*(6), 1117-1124. doi:10.1007/s11250-017-1299-3

Stankevicius, A, Buitkuviene, J, Deltuvytiene, J, Cepuliene, R, Zilionyte, V, Pampariene, I, Zymantiene, J (2014) Five Years Seroprevalence Study of Porcine Reproductive and Respiratory Syndrome Virus in Lithuanian Pig and Wild Boar Populations. *Bulletin- Veterinary Institute in Pulawy, 58*, 379-383. doi:10.2478/bvip-2014-0059

Štukelj, M, Toplak, I, Vengušt, G (2014) Prevalence of Antibodies against Selected Pathogens in Wild Boars (Sus Scrofa) in Slovenia. *Slovenian Veterinary Research, 51*(1), 21-28.

Touloudi, A, Valiakos, G, Athanasiou, LV, Birtsas, P, Giannakopoulos, A, Papaspyropoulos, K, Kalaitzis, C, Sokos, C, Tsokana, CN, Spyrou, V, Petrovska, L, Billinis, C (2015) A Serosurvey for Selected Pathogens in Greek European Wild Boar. *Veterinary Record Open, 2*(2), e000077. doi:10.1136/vetreco-2014-000077

Tummaruk, P, Tantilertcharoen, R (2012) Seroprevalence of Porcine Reproductive and Respiratory Syndrome, Aujeszky's Disease, and Porcine Parvovirus in Replacement Gilts in Thailand. *Tropical Animal Health and Production, 44*(5), 983-989. doi:10.1007/s11250-011-9999-6

Vengust, G, Valencak, Z, Bidovec, A (2006) A Serological Survey of Selected Pathogens in Wild Boar in Slovenia. *Journal of Veterinary Medicine, Series B, 53*(1), 24-27. doi:10.1111/j.1439-0450.2006.00899.x

Vicente, J, León-Vizcaíno, L, Gortázar, C, Cubero, MJ, González, M, Martín-Atance, P (2002) Antibodies to Selected Viral and Bacterial Pathogens in European Wild Boars from Southcentral Spain. *Journal of Wildlife Diseases, 38*(3), 649-652. doi:10.7589/0090-3558-38.3.649

Wayne, SR, Morrison, RB, Odland, CA, Davies, PR (2012) Potential Role of Noncommercial Swine Populations in the Epidemiology and Control of Porcine Reproductive and Respiratory Syndrome Virus. *Journal of the American Veterinary Medical Association, 240*(7), 876-882. doi:10.2460/javma.240.7.876

Wiratsudakul, A, Prompiram, P, Poltep, K, Tantawet, S, Suraruangchai, D, Sedwisai, P, Sangkachai, N, Ratanakorn, P (2013) A Cross-Sectional Study of Porcine Reproductive and Respiratory Syndrome Virus and Mycoplasma Hyopneumoniae in Wild Boars Reared in Different Types of Captive Setting in Thailand. *Journal of Veterinary Science & Technology, 04*. doi:10.4172/2157-7579.1000146

Wu, N, Abril, C, Hinić, V, Brodard, I, Thür, B, Fattebert, J, Hüssy, D, Ryser-Degiorgis, MP (2011) Free-Ranging Wild Boar: A Disease Threat to Domestic Pigs in Switzerland? *Journal of Wildlife Diseases, 47*(4), 868-879. doi:10.7589/0090-3558-47.4.868

Wyckoff, AC, Henke, SE, Campbell, TA, Hewitt, DG, VerCauteren, KC (2009) Feral Swine Contact with Domestic Swine: A Serologic Survey and Assessment of Potential for Disease Transmission. *Journal of Wildlife Diseases, 45*(2), 422-429. doi:10.7589/0090-3558-45.2.422

Yu, H, Zhang, L, Cai, Y, Hao, Z, Luo, Z, Peng, T, Liu, L, Wang, N, Wang, G, Deng, Z, Zhan, Y (2022) Seroprevalence of Antibodies to Classical Swine Fever Virus and Porcine Reproductive and Respiratory Syndrome Virus in Healthy Pigs in Hunan Province, China. *Polish Journal of Veterinary Sciences, 25*(3), 375-381. doi:10.24425/pjvs.2022.142020

Zhao, P, Wang, C, Cao, W, Fang, R, Zhao, J (2022) Risk Factors and Spatial-Temporal Analysis of Porcine Reproductive and Respiratory Syndrome Seroprevalence in China before and after African Swine Fever Outbreak. *Frontiers in Veterinary Science, 9*, 929596. doi:10.3389/fvets.2022.929596

Zupancić, Z, Jukić, B, Lojkić, M, Cac, Z, Jemersić, L, Staresina, V (2002) Prevalence of Antibodies to Classical Swine Fever, Aujeszky's Disease, Porcine Reproductive and Respiratory Syndrome, and Bovine Viral Diarrhoea Viruses in Wild Boars in Croatia. *Journal of Veterinary Medicine Series B-Infectious Diseases and Veterinary Public Health, 49*(5), 253-256. doi:10.1046/j.1439-0450.2002.00562.x
