# Supplementary material for: A Systematic Review and Meta-Analysis on the Global Seroprevalence of Porcine Reproductive and Respiratory Virus (PRRSV) in Pigs and Wild Boars: A Widespread and Impactful Swine Virus
Source: Vet Sci. 2026 Mar 23;13(3):304. doi: 10.3390/vetsci13030304 (PMC13030171; doi:10.3390/vetsci13030304)
Supplement: Supplementary file 1 [file vetsci-13-00304-s001.zip › Supplementary_Material_1.pdf]

Supplementary Material S1: Systematic review and Meta-Analysis Protocol (PRISMA-P)

ADMINISTRATIVE INFORMATION

Title:

Serological prevalence of porcine reproductive and respiratory syndrome virus in domestic pigs and wild boars on a global scale: protocol for a systematic review

Authors

Graziosi G.[1], Longobardi C.[2], Lupini C.[1], Catelli E.[1], Ferrara G.[3]

[1]Department of Veterinary Medical Sciences, University of Bologna, 40064 Ozzano Emilia (BO), Italy;

[2]Department of Veterinary Medicine and Animal production, University of Naples, Federico II, 80137 Naples, Italy;

[3]Department of Veterinary Sciences, University of Messina, 98168 Messina, Italy

Contacts

Graziosi G.: giulia.graziosi2@unibo.it

Longobardi C.: consiglia.longobardi@unina.it

Lupini C.: caterina.lupini@unibo.it

Catelli E.: elena.catelli@unibo.it

Ferrara G.: g.ferrara@unime.it

Contributions

Conceptualisation, G.G. and G.F.; acquisition of data, G.G., C.L., Ca.L., E.C., G.F.; statistical analysis, G.G.; interpretation of data, G.G. and C.L.; writing – original draft preparation, G.G.; writing – review and editing, G.G., C.L., Ca.L., E.C. and G.F.; supervision, G.F. All authors have read and agreed to the published version of the manuscript.

Funding sources/sponsors

No external funding sought.

INTRODUCTION

Rationale

Porcine reproductive and respiratory syndrome virus (PRRSV) is considered one of the most economically important swine diseases worldwide. The frequency of infection is often high in affected nations and is proportional to animal density. Considering the absence of global seroprevalence data in swine and wild boars, we carry out a systematic review and meta-analysis to assess the seroprevalence of PRRSV in suids.

Objectives

The aim of this study is to assess the global seroprevalence of PRRSV by reviewing the articles in the literature and identifying any risk variables associated with greater exposures.

METHODS

Eligibility criteria

The following PICO was applied: studies conducted in domestic pigs (*Sus scrofa domesticus*) and wild boars (*Sus scrofa* (Population) evaluating the natural infection of PRRSV (Intervention; Comparator, not applicable). An article was considered eligible if the following requirements were met: 1) the study reported information on the prevalence of PRSSV in domestic pigs or wild boars; 2) a serological test was used to assess exposure to PRSSV.

Information sources

Literature searches are carried out of the following repositories: PubMed, Scopus and Web of Sciences.

Dates of coverage: November 2024 – June 2025

Search strategy

The following PICO was applied: studies conducted in domestic pigs (*Sus scrofa domesticus*) and wild boars (*Sus scrofa* (Population) evaluating the natural infection of PRRSV (Intervention; Comparator, not applicable). The included studies measured and reported the positivity rate or prevalence of PRSSV (Outcome). Search strategy included the following concept following PICO: ‘porcine respiratory and reproductive syndrome’ AND ‘antibody’ AND ‘wild boars’ OR ‘swine’

1. Serological studies on PRRSV, search lines.

Database	Search line	No. of studies retrieved

**PubMed**	(("porcine reproductive and respiratory syndrome"[Title/Abstract] OR PRRSV[Title/Abstract] OR "porcine reproductive and respiratory syndrome virus"[All Fields]) OR ("arterivirus infections"[MeSH Terms] OR "arteriviridae"[MeSH Terms])) AND ("serologic tests"[MeSH Terms] OR serology[Title/Abstract] OR serological[Title/Abstract] OR "antibodies, viral"[MeSH Terms] OR antibody[Title/Abstract] OR antibodies[Title/Abstract]) AND (("swine"[MeSH Terms] OR pigs[Title/Abstract]) OR ("sus scrofa"[MeSH Terms] OR (wild[Title/Abstract] AND (boars[Title/Abstract] OR swine[Title/Abstract]))))	1,372
**Scopus**	(TITLE-ABS ("porcine reproductive and respiratory syndrome virus") OR TITLE-ABS (PRRSV) OR TITLE-ABS ("porcine reproductive and respiratory syndrome")) AND (TITLE-ABS (serolog*) OR TITLE-ABS (antibody) OR TITLE-ABS (antibodies) OR TITLE-ABS (serum)) AND (TITLE-ABS (swine) OR TITLE-ABS (pigs) OR (TITLE-ABS (wild) AND (TITLE-ABS (boar*) OR TITLE-ABS (pig*) OR TITLE-ABS ("Sus scrofa"))))	1,518
**Web of Science**	TS=("porcine reproductive and respiratory syndrome virus" OR PRRSV OR "porcine reproductive and respiratory syndrome") AND TS=(serology OR serological OR antibody OR antibodies OR serum) AND TS=("wild boar" OR "Sus scrofa" OR swine OR pigs) AND TS=(detection OR prevalence)	513

Study records

Data management, selection process and data collection process

Data extraction was performed by five independent reviewers and the quality of the data was double checked by a third author. If data retrieved from original articles were expressed as percentages, raw number were obtained converting the percentages to the closer integer.

One data extraction sheet was created on Microsoft Excel 2021 (version 16.49). The following information were annotated: first author, year of the publication, title, country, region, sampling period, host (domestic pig or wild boar), age classes and sex, housing, number of animals sampled, number of animals testing positive and diagnostic method applied. If an object is not provided, 'N.R.' (NOT REPORTED) is written. If some data are difficult to extract, a comment is written in that cell.

Data items – seroprevalence

**Variable**	**Explanation**
**Title**	Write the complete title
**First author**	Write the last name of the first author
**Year of publication**	Write the year when the study was published in the journal or take the date from the respective source (i.e. conference abstracts)
**Country**	Write the country where samples were collected
**Sampling period**	Write the years during which the samples were collected
**Host identification**	Write the species
**Age classes**	Write the age classes of the animals tested
**Sex**	Write the sex of the animals tested
**Housing**	Write the housing system of the animals tested
**Total number of animals (N)**	Write the total number of animals tested in the study

**Total number of cases (n)**	Write the total number of seropositive animals
**Serological method**	Write the type of the serologic test used
**Other comments**	Write any comment relevant to interpret the seroprevalence

If the object is not provided, 'N.R.' (NOT REPORTED) is written. If some data are difficult to extract, a comment is written in that cell.

Primary outcomes

Estimate of the sero-prevalence of PRRSV in domestic pigs and wild boars without any geographic restriction (positive cases / total number of animals tested).

Additional outcomes

Qualitative analyses of categorical variables related to the wild hosts as possibly relevant to further epidemiological considerations (species, country, method, sex).

Risk of bias in individual studies

To minimize the risk of bias in individual studies, anything that could potentially affect the interpretation of the seroprevalence will be written in the comment section of data extraction.

Data synthesis

If studies are sufficiently homogeneous, a meta-analysis using a random-effects model is carried out and the inverse variance index I^2 is used to quantify heterogeneity as low (I^2 <25%), moderate (I^2: 25%-75%) and high (I^2 >75%) heterogeneity.

Subgroup analyses according to species, country, age and sex of domestic pigs and wild boars could be performed if data are consistent.

Deviations from protocol

No deviations from this protocol were reported.

PRISMA-P (Preferred Reporting Items for Systematic review and Meta-Analysis Protocols) 2020 checklist: recommended items to address in a systematic review protocol

Section and Topic	Item #	Checklist item	Location where item is reported
**TITLE**			
Title	1	Identify the report as a systematic review.	p.1
**ABSTRACT**			
Abstract	2	See the PRISMA 2020 for Abstracts checklist.	p.1
**INTRODUCTION**			
Rationale	3	Describe the rationale for the review in the context of existing knowledge.	p.1
Objectives	4	Provide an explicit statement of the objective(s) or question(s) the review addresses.	p.2
**METHODS**			
Eligibility criteria	5	Specify the inclusion and exclusion criteria for the review and how studies were grouped for the syntheses.	p.4
Information sources	6	Specify all databases, registers, websites, organisations, reference lists and other sources searched or consulted to identify studies. Specify the date when each source was last searched or consulted.	p.3-4
Search strategy	7	Present the full search strategies for all databases, registers and websites, including any filters and limits used.	Table 1

Section and Topic	Item #	Checklist item	Location where item is reported
Selection process	8	Specify the methods used to decide whether a study met the inclusion criteria of the review, including how many reviewers screened each record and each report retrieved, whether they worked independently, and if applicable, details of automation tools used in the process.	p.4
Data collection process	9	Specify the methods used to collect data from reports, including how many reviewers collected data from each report, whether they worked independently, any processes for obtaining or confirming data from study investigators, and if applicable, details of automation tools used in the process.	p.4
Data items	10a	List and define all outcomes for which data were sought. Specify whether all results that were compatible with each outcome domain in each study were sought (e.g. for all measures, time points, analyses), and if not, the methods used to decide which results to collect.	p.4
	10b	List and define all other variables for which data were sought (e.g. participant and intervention characteristics, funding sources). Describe any assumptions made about any missing or unclear information.	p.4
Study risk of bias assessment	11	Specify the methods used to assess risk of bias in the included studies, including details of the tool(s) used, how many reviewers assessed each study and whether they worked independently, and if applicable, details of automation tools used in the process.	p.4-5
Effect measures	12	Specify for each outcome the effect measure(s) (e.g. risk ratio, mean difference) used in the synthesis or presentation of results.	p.5
Synthesis	13a	Describe the processes used to decide which studies were eligible for each synthesis (e.g. tabulating the study	p.4

Section and Topic	Item #	Checklist item	Location where item is reported
methods		intervention characteristics and comparing against the planned groups for each synthesis (item #5)).	
	13b	Describe any methods required to prepare the data for presentation or synthesis, such as handling of missing summary statistics, or data conversions.	p.4
	13c	Describe any methods used to tabulate or visually display results of individual studies and syntheses.	p.4
	13d	Describe any methods used to synthesize results and provide a rationale for the choice(s). If meta-analysis was performed, describe the model(s), method(s) to identify the presence and extent of statistical heterogeneity, and software package(s) used.	p.5
	13e	Describe any methods used to explore possible causes of heterogeneity among study results (e.g. subgroup analysis, meta-regression).	p.5
	13f	Describe any sensitivity analyses conducted to assess robustness of the synthesized results.	Not applicable
Reporting bias assessment	14	Describe any methods used to assess risk of bias due to missing results in a synthesis (arising from reporting biases).	Not applicable
Certainty assessment	15	Describe any methods used to assess certainty (or confidence) in the body of evidence for an outcome.	Not applicable
**RESULTS**			
Study	16a	Describe the results of the search and selection process, from the number of records identified in the search to the	p.5-6

Section and Topic	Item #	Checklist item	Location where item is reported
selection		number of studies included in the review, ideally using a flow diagram.	
	16b	Cite studies that might appear to meet the inclusion criteria, but which were excluded, and explain why they were excluded.	Figure 1
Study characteristics	17	Cite each included study and present its characteristics.	Table 2
Risk of bias in studies	18	Present assessments of risk of bias for each included study.	Not applicable
Results of individual studies	19	For all outcomes, present, for each study: (a) summary statistics for each group (where appropriate) and (b) an effect estimate and its precision (e.g. confidence/credible interval), ideally using structured tables or plots.	Figure 2
Results of syntheses	20a	For each synthesis, briefly summarise the characteristics and risk of bias among contributing studies.	Not applicable
	20b	Present results of all statistical syntheses conducted. If meta-analysis was done, present for each the summary estimate and its precision (e.g. confidence/credible interval) and measures of statistical heterogeneity. If comparing groups, describe the direction of the effect.	p.6
	20c	Present results of all investigations of possible causes of heterogeneity among study results.	p.6
	20d	Present results of all sensitivity analyses conducted to assess the robustness of the synthesized results.	Not

Section and Topic	Item #	Checklist item	Location where item is reported
			applica-ble
Reporting bi-ases	21	Present assessments of risk of bias due to missing results (arising from reporting biases) for each synthesis assessed.	Not appli-cable
Certainty of evidence	22	Present assessments of certainty (or confidence) in the body of evidence for each outcome assessed.	Not appli-cable
**DISCUSSION**			
Discussion	23a	Provide a general interpretation of the results in the context of other evidence.	p.7
	23b	Discuss any limitations of the evidence included in the review.	p.8
	23c	Discuss any limitations of the review processes used.	p.8-9
	23d	Discuss implications of the results for practice, policy, and future research.	p.8-9
**OTHER INFORMATION**			
Registration and protocol	24a	Provide registration information for the review, including register name and registration number, or state that the re-view was not registered.	Supple-mentary Materials 1

Section and Topic	Item #	Checklist item	Location where item is reported
	24b	Indicate where the review protocol can be accessed, or state that a protocol was not prepared.	Supple-mentary Materials 1
	24c	Describe and explain any amendments to information provided at registration or in the protocol.	Not appli-cable
Support	25	Describe sources of financial or non-financial support for the review, and the role of the funders or sponsors in the review.	p.8
Competing in-terests	26	Declare any competing interests of review authors.	p.9
Availability of data, code and other materials	27	Report which of the following are publicly available and where they can be found: template data collection forms; data extracted from included studies; data used for all analyses; analytic code; any other materials used in the review.	p.3; p.5

From: Page MJ, McKenzie JE, Bossuyt PM, Boutron I, Hoffmann TC, Mulrow CD, et al. The PRISMA 2020 statement: an updated guideline for reporting systematic reviews. BMJ 2021;372:n71. doi: 10.1136/bmj.n71

For more information, visit: http://www.prisma-statement.org/
