# Supplementary material for: A Systematic Review and Meta-Analysis on the Global Seroprevalence of Porcine Reproductive and Respiratory Virus (PRRSV) in Pigs and Wild Boars: A Widespread and Impactful Swine Virus
Source: Vet Sci. 2026 Mar 23;13(3):304. doi: 10.3390/vetsci13030304 (PMC13030171; doi:10.3390/vetsci13030304)
Supplement: Supplementary file 1 [file vetsci-13-00304-s001.zip › Supplementary_Material_3.pdf]

Supplementary Material S3: Outliers identification for seroprevalence studies on PRRSV in suids.

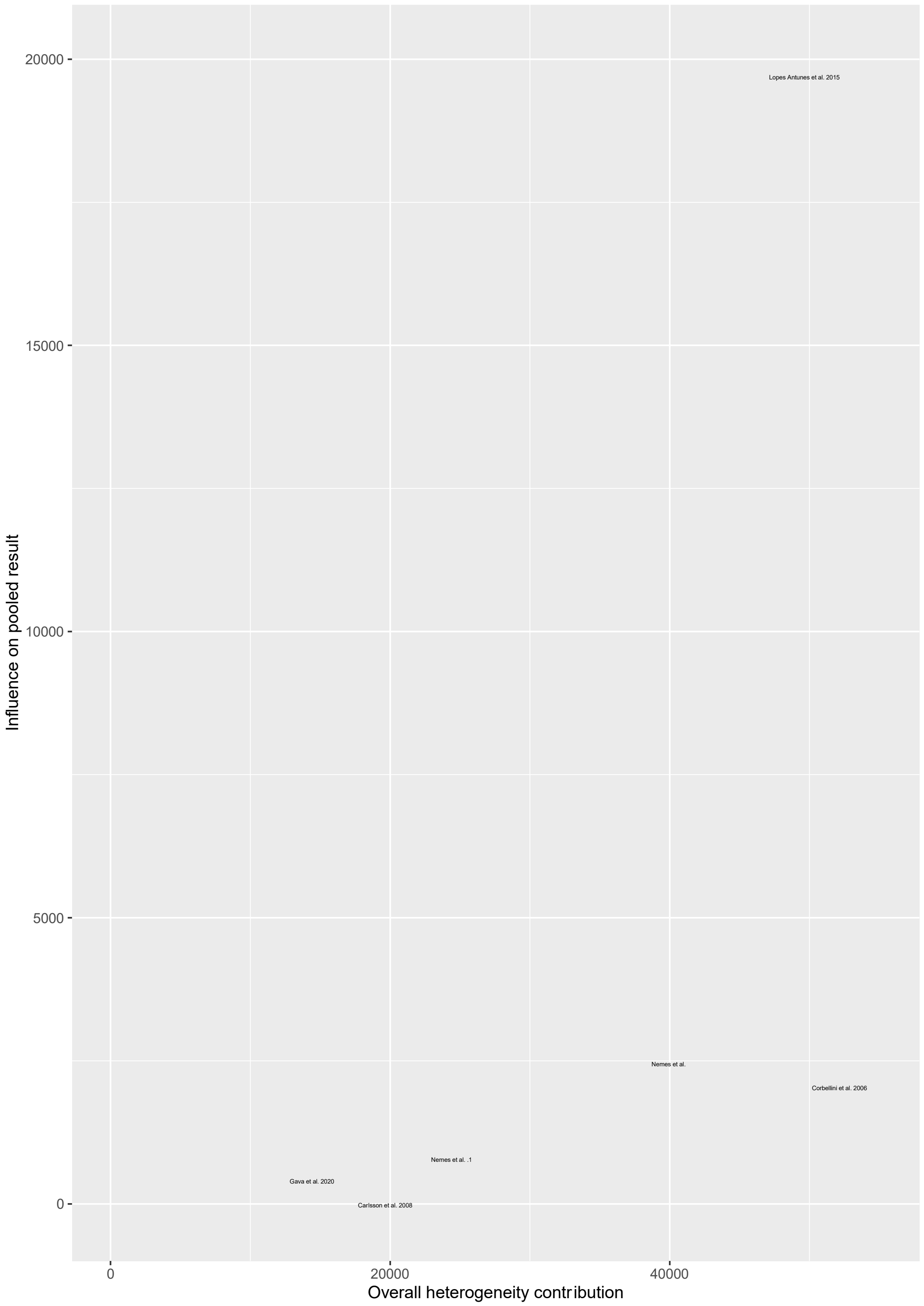

Figure A1: The Baujat Plot. The study conducted by Antunes et al. (2015); Corbellini et al. (2006); Nemes et al. (2019) could be identified as outliers which may influence the summary effect size estimate [10,17,28,47,59].

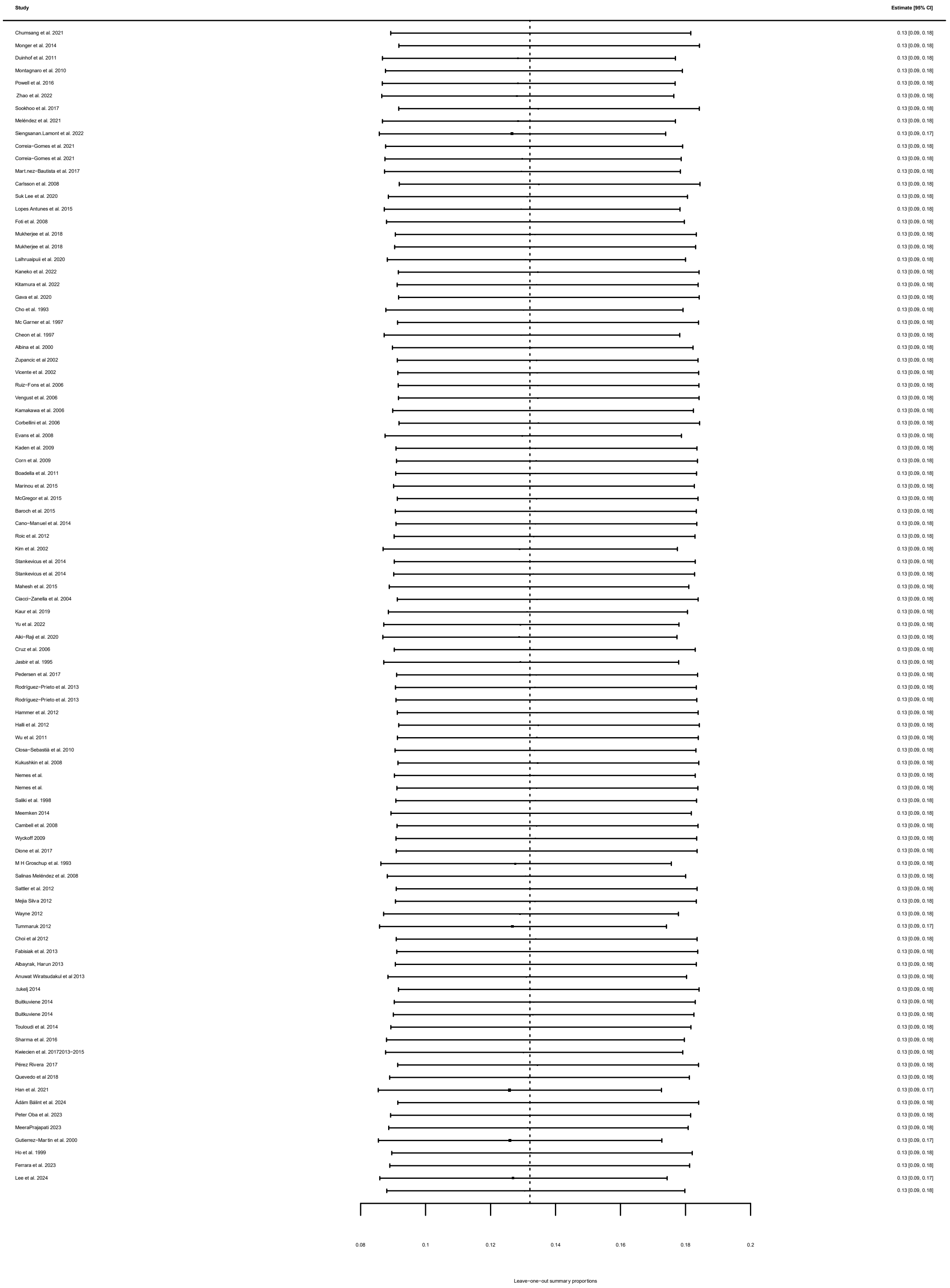

Figure A2: Leave-one out analysis on the studies reporting seroprevalence of PRRSV in suids. Each box represents a summary proportion estimated leaving out a study. The vertical dotted line indicates where the original pooled prevalence lies (14%, 95% C.I.: 9%-19%) [1-86].

References

1. Aiki-Raji, C, Adebiyi, A, Abiola, O, Oluwayelu, D (2018) Prevalence of Porcine Reproductive and Respiratory Syndrome Virus and Porcine Parvovirus Antibodies in Commercial Pigs, Southwest Nigeria. *Beni-Suef University Journal of Basic and Applied Sciences, 7*. doi:10.1016/j.bjbas.2017.07.006
2. Albayrak, H, Ozan, E, Cavunt, A (2013) A Serological Survey of Selected Pathogens in Wild Boar (Sus Scrofa) in Northern Turkey. *European Journal of Wildlife Research, 59*(6), 893-897. doi:10.1007/s10344-013-0743-6
3. Albina, E, Mesplède, A, Chenut, G, Le Potier, MF, Bourbao, G, Le Gal, S, Leforban, Y (2000) A Serological Survey on Classical Swine Fever (Csf), Aujeszky's Disease (Ad) and Porcine Reproductive and Respiratory Syndrome (Prrs) Virus Infections in French Wild Boars from 1991 to 1998. *Veterinary Microbiology, 77*(1), 43-57. doi:10.1016/S0378-1135(00)00255-8
4. Bálint, Á, Csányi, S, Nemes, I, Bijl, H, Szabó, I (2024) Investigation of Prrs Virus Infection in Hungarian Wild Boar Populations During Its Eradication from Domestic Pig Herds. *Animals, 14*(11), 1537. doi:10.3390/ani14111537
5. Baroch, JA, Gagnon, CA, Lacouture, S, Gottschalk, M (2015) Exposure of Feral Swine (Sus Scrofa) in the United States to Selected Pathogens. *Canadian Journal of Veterinary Research, 79*(1), 74-78.
6. Boadella, M, Ruiz-Fons, JF, Vicente, J, Martín, M, Segalés, J, Gortazar, C (2012) Seroprevalence Evolution of Selected Pathogens in Iberian Wild Boar. *Transboundary and Emerging Diseases, 59*(5), 395-404. doi:10.1111/j.1865-1682.2011.01285.x
7. Buitkuvienė, J, Deltuvytienė, J, Čepulienė, R, Žilionytė, V, Mozūraitytė, J, Pridotkas, G, Stankevičius, A (2014) Serological Survey on Porcine Reproductive and Respiratory Syndrome Virus (Prrsv) in Lithuanian Pigs and Wild Boars. *Veterinarija ir Zootechnika, 67*(89).
8. Campbell, TA, DeYoung, RW, Wehland, EM (2008) Feral Swine Exposure to Selected Viral and Bacterial Pathogens in Southern Texas. *JSHAP, 16*(6), 312-315.
9. Cano-Manuel, FJ, López-Olvera, J, Fandos, P, Soriguer, RC, Pérez, JM, Granados, JE (2014) Long-Term Monitoring of 10 Selected Pathogens in Wild Boar (Sus Scrofa) in Sierra Nevada National Park, Southern Spain. *Veterinary Microbiology, 174*(1), 148-154. doi:10.1016/j.vetmic.2014.06.017
10. Carlsson, U, Wallgren, P, Renström, LHM, Lindberg, A, Eriksson, H, Thorén, P, Eliasson-Selling, L, Lundeheim, N, Nörregard, E, Thörn, C, Elvander, M (2009) Emergence of Porcine Reproductive and Respiratory Syndrome in Sweden: Detection, Response and Eradication. *Transboundary and Emerging Diseases, 56*(4), 121-131. doi:10.1111/j.1865-1682.2008.01065.x
11. Cheon, D-S, Chae, C, Lee, Y-S (1997) Seroprevalence of Antibody to Porcine Reproductive and Respiratory Syndrome Virus Using Enzyme-Linked Immunosorbent Assay in Selected Herds in Korea. *Journal of Veterinary Diagnostic Investigation, 9*(4), 434-436. doi:10.1177/104063879700900419
12. Cho, SH, Freese, WR, Yoon, IJ, Trigo, AV, Joo, HS (1993) Seroprevalence of Indirect Fluorescent Antibody to Porcine Reproductive and Respiratory Syndrome Virus in Selected Swine Herds. *Journal of Veterinary Diagnostic Investigation, 5*(2), 259-260. doi:10.1177/104063879300500220
13. Choi, E-J, Lee, C-H, Hyun, B-H, Kim, J-J, Lim, S-I, Song, J-Y, Shin, Y-K (2012) A Survey of Porcine Reproductive and Respiratory Syndrome among Wild Boar Populations in Korea. *Journal of Veterinary Science, 13*(4), 377-383. doi:10.4142/jvs.2012.13.4.377
14. Chumsang, S, Na Lampang, K, Srikitjakarn, L, Pringproa, K (2021) Seroprevalence of the Viral Pig Diseases among Backyard Pigs in Chiang Mai, Thailand. *Preventive Veterinary Medicine, 190*, 105330. doi:10.1016/j.prevetmed.2021.105330

15. Ciacci-Zanella, JR, Trombetta, C, Vargas, I, Mariano da Costa, DE (2004) Lack of Evidence of Porcine Reproductive and Respiratory Syndrome Virus (Prrsv) Infection in Domestic Swine in Brazil. *Ciência Rural, 32*(2), 449–455.
16. Closa-Sebastià, F, Casas-Díaz, E, Cuenca, R, Lavín, S, Mentaberre, G, Marco, I (2011) Antibodies to Selected Pathogens in Wild Boar (Sus Scrofa) from Catalonia (Ne Spain). *European Journal of Wildlife Research, 57*(4), 977-981. doi:10.1007/s10344-010-0491-9
17. Corbellini, LG, Schwermer, H, Presi, P, Thür, B, Stärk, KDC, Reist, M (2006) Analysis of National Serological Surveys for the Documentation of Freedom from Porcine Reproductive and Respiratory Syndrome in Switzerland. *Veterinary Microbiology, 118*(3), 267-273. doi:10.1016/j.vetmic.2006.07.018
18. Corn, JL, Cumbee, JC, Barfoot, R, Erickson, GA (2009) Pathogen Exposure in Feral Swine Populations Geographically Associated with High Densities of Transitional Swine Premises and Commercial Swine Production. *Journal of Wildlife Diseases, 45*(3), 713-721. doi:10.7589/0090-3558-45.3.713
19. Correia-Gomes, C, Duncan, A, Ward, A, Pearce, M, Eppink, L, Webster, G, McGowan, A, Thomson, J (2022) Porcine Reproductive and Respiratory Syndrome Virus Seroprevalence in Scottish Finishing Pigs between 2006 and 2018. *Veterinary Record, 190*(7), e349. doi:10.1002/vetr.349
20. Cruz, MC (2006) Prevalencia Serológica Del Síndrome Reproductivo Y Respiratorio Porcino (Prrs) En Cerdos De Explotaciones Extensivas De Colombia. *Rev Med Vet Zoot., 53*, 33-41.
21. Dione, M, Masembe, C, Akol, J, Amia, W, Kungu, J, Lee, HS, Wieland, B (2018) The Importance of on-Farm Biosecurity: Sero-Prevalence and Risk Factors of Bacterial and Viral Pathogens in Smallholder Pig Systems in Uganda. *Acta Tropica, 187*, 214-221. doi:10.1016/j.actatropica.2018.06.025
22. Duinhof, TF, van Schaik, G, van Esch, EJB, Wellenberg, GJ (2011) Detection of Prrsv Circulation in Herds without Clinical Signs of Prrs: Comparison of Five Age Groups to Assess the Preferred Age Group and Sample Size. *Veterinary Microbiology, 150*(1), 180-184. doi:10.1016/j.vetmic.2011.01.001
23. Evans, CM, Medley, GF, Green, LE (2008) Porcine Reproductive and Respiratory Syndrome Virus (Prrsv) in Gb Pig Herds: Farm Characteristics Associated with Heterogeneity in Seroprevalence. *BMC Veterinary Research, 4*(1), 48. doi:10.1186/1746-6148-4-48
24. Fabisiak, M, Podgórska, K, Skrzypiec, E, Szczotka, A, Stadejek, T (2013) Detection of Porcine Circovirus Type 2 (Pcv2) and Porcine Reproductive and Respiratory Syndrome Virus (Prrsv) Antibodies in Meat Juice Samples from Polish Wild Boar (Sus Scrofa L.). *Acta Veterinaria Hungarica, 61*(4), 529-536. doi:10.1556/avet.2013.027
25. Ferrara, G, D'Anza, E, Rossi, A, Improda, E, Iovane, V, Pagnini, U, Iovane, G, Montagnaro, S (2023) A Serological Investigation of Porcine Reproductive and Respiratory Syndrome and Three Coronaviruses in the Campania Region, Southern Italy. *Viruses, 15*(2), 300.
26. Foti, M, Bottari, T, Daidone, A, Rinaldo, D, De Leo, F, Foti, S, Giacopello, C (2008) Serological Survey on Aujeszky's Disease, Swine Influenza and Porcine Reproductive and Respiratory Syndrome Virus Infections in Italian Pigs. *Polish Journal of Veterinary Sciences, 11*(4), 323-325.
27. Garner, MG, Gleeson, LJ, Holyoake, PK, Cannon, RM, Doughty, WJ (1997) A National Serological Survey to Verify Australia's Freedom from Porcine Reproductive and Respiratory Syndrome. *Australian Veterinary Journal, 75*(8), 596-600. doi:10.1111/j.1751-0813.1997.tb14202.x
28. Gava, D, Caron, L, Schaefer, R, Silva, VS, Weiblen, R, Flores, EF, de Lima, M, Takeda, GZ, Ciacci-Zanella, JR (2022) A Retrospective Study of Porcine Reproductive and Respiratory Syndrome Virus Infection in Brazilian Pigs from 2008 to 2020. *Transboundary and Emerging Diseases, 69*(2), 903-907. doi:10.1111/tbed.14036

29. Groschup, MH, Brun, A, Haas, B (1993) Serological Studies on the Potential Synergism of Porcine Reproductive and Respiratory Syndrome Virus and Influenza-, Corona- and Paramyxoviruses in the Induction of Respiratory Symptoms in Swine. *Journal of Veterinary Medicine, Series B, 40*(1-10), 681-689. doi:10.1111/j.1439-0450.1993.tb00192.x
30. Gutiérrez-Martín, CB, RodrÍguez-Delgado, Ó, ÁLvarez-Nistal, D, De La Puente-Redondo, VA, GarcÍa-Rioja, F, MartÍn-Vicente, J, RodrÍguez Ferri, EF (2000) Simultaneous Serological Evidence of Actinobacillus Pleuropneumoniae, Prrs, Aujeszky's Disease and Influenza Viruses in Spanish Finishing Pigs. *Research in Veterinary Science, 68*(1), 9-13. doi:10.1053/rvsc.1999.0326
31. Hälli, O, Ala-Kurikka, E, Nokireki, T, Skrzypczak, T, Raunio-Saarnisto, M, Peltoniemi, OA, Heinonen, M (2012) Prevalence of and Risk Factors Associated with Viral and Bacterial Pathogens in Farmed European Wild Boar. *The Veterinary Journal, 194*(1), 98-101. doi:10.1016/j.tvjl.2012.03.008
32. Hammer, R, Ritzmann, M, Palzer, A, Lang, C, Hammer, B, Pesch, S, Ladinig, A (2012) Porcine Reproductive and Respiratory Syndrome Virus and Porcine Circovirus Type 2 Infections in Wild Boar (Sus Scrofa) in Southwestern Germany. *Journal of Wildlife Diseases, 48*(1), 87-94. doi:10.7589/0090-3558-48.1.87
33. Han, D, Yang, H, Yang, Y, Ye, L, Dong, J, Zhang, C, Zhu, H, Yin, S, Dong, X, Su, F, Xin, J, Ai, J (2021) Porcine Reproductive and Respiratory Syndrome Virus (Prrsv) Antibody Levels in Large Swine Farms in Selected Regions of Yunnan Province, China. *Medycyna Weterynaryjna, 77*, 497-501. doi:10.21521/mw.6581
34. Ho, S-h, Jun, Y-c, Park, C-k, Lee, C-h, Bae, J-h (1999) Prevalence of Tissue Antigen and Serum Antibody for Porcine Reproductive and Respiratory Syndrome in Cheju. *Korean Journal of Veterinary Research, 39*(4), 760-764.
35. Jasbir, S, Hussin, AA, Arunasalam, V (1995) Seroprevalence of Porcine Reproductive and Respiratory Syndrome (Prrs). *Malaysian Journal of Veterinary Research, 7*, 81-82.
36. Kaden, V, Lange, E, Hänel, A, Hlinak, A, Mewes, L, Hergarten, G, Irsch, B, Dedek, J, Bruer, W (2009) Retrospective Serological Survey on Selected Viral Pathogens in Wild Boar Populations in Germany. *European Journal of Wildlife Research, 55*(2), 153-159. doi:10.1007/s10344-008-0229-0
37. Kamakawa, A, Ho, TV, Yamada, S (2006) Epidemiological Survey of Viral Diseases of Pigs in the Mekong Delta of Vietnam between 1999 and 2003. *Veterinary Microbiology, 118*(1-2), 47-56. doi:10.1016/j.vetmic.2006.07.003
38. Kaneko, F, Kitamura, N, Suzuki, K, Kato, M (2022) Serological Survey of Antibodies to Four Pathogens in Wild Boars in Nagano Prefecture, Japan. *Journal of Veterinary Medical Science, 84*(6), 855-859. doi:10.1292/jvms.22-0035
39. Kaur, A, Mahajan, V, Leishangthem, G, Singh, N, Banga, H, Filia, G (2019) Seroprevalence Study for Detection of Porcine Reproductive and Respiratory Syndrome Virus Antibodies in Pig Population of Punjab. *Haryana Veterinarian, 58*(1), 122–123. doi:10.14202/vetworld.2016.827-831
40. Kim, SM, Han, TU, Kang, SY, Shin, KS, Kim, CJ, Kim, JT, Kim, HS (2002) Seroprevalence of Antibody to Procine Reproductive and Respiratory Syndrome Virus in Diagnostic Submissions. *Journal of Veterinary Science, 3*(3), 159-161.
41. Kitamura, Y, Saito, T, Tanaka, E, Takashima, Y (2022) A Serological Survey of Porcine Reproductive and Respiratory Syndrome Virus in Wild Boar in Gifu Prefecture, Japan. *Journal of Veterinary Medical Science, 84*(10), 1406-1409. doi:10.1292/jvms.21-0554
42. Kukushkin, S, Kanshina, A, Timina, A, Baybikov, T, Mikhalishin, V (2008) Investigation of Wild Boar (Sus Scrofa) for Porcine Reproductive and Respiratory Syndrome in Some Territories of Russia. *European Journal of Wildlife Research, 54*(3), 515-518. doi:10.1007/s10344-007-0159-2

43. Kwiecien, EJ, Mejía-Silva, W, Quintero-Moreno, A, Gutierrez, C (2017) Estudio De La Respuesta Serológica Contra El Virus Del Síndrome Reproductivo Y Respiratorio Porcino (Prrs) En Cerdos Bajo Condiciones Tropicales. *Revista Científica, 27*(5), 282-293.
44. Lalhruaipuii, K, Shakuntala, I, Sen, A (2020) Seroprevalence of Porcine Reproductive and Respiratory Syndrome Virus and Classical Swine Fever Virus in Pigs of Mizoram, India. *Journal of Environmental Biology, 41*, 915-920. doi:10.22438/jeb/4(SI)/MS_1920
45. Lee, HS, Bui, VN, Nguyen, HX, Bui, AN, Hoang, TD, Nguyen-Viet, H, Grace Randolph, D, Wieland, B (2020) Seroprevalences of Multi-Pathogen and Description of Farm Movement in Pigs in Two Provinces in Vietnam. *BMC Veterinary Research, 16*(1), 15. doi:10.1186/s12917-020-2236-7
46. Lee, YB, Kim, JW, Jo, W, Kang, TK, Sung, M, Kim, K, Park, NH, Lee, GH (2024) Assessment of Prrsv and Pcv2 Seroprevalence and Antigen Prevalence in Minipigs at Laboratory-Animal Production Facilities. *Journal of Advanced Veterinary and Animal Research, 11*(4), 1017-1022. doi:10.5455/javar.2024.k852
47. Lopes Antunes, AC, Halasa, T, Lauritsen, KT, Kristensen, CS, Larsen, LE, Toft, N (2015) Spatial Analysis and Temporal Trends of Porcine Reproductive and Respiratory Syndrome in Denmark from 2007 to 2010 Based on Laboratory Submission Data. *BMC Veterinary Research, 11*(1), 303. doi:10.1186/s12917-015-0617-0
48. Mahesh, K, Bhoj, J, Swoyam, S, Meera, P, Dipak, K, Santosh, D (2015) Sero-Prevalence of Porcine Reproductive and Respiratory Syndrome (Prrs) in Pigs of Different Developmental Regions of Nepal. *International journal of applied sciences and biotechnology, 3*, 218-222. doi:10.3126/ijasbt.v3i2.12539
49. Marinou, KA, Papatsiros, VG, Gkotsopoulos, EK, Odatzoglou, PK, Athanasiou, LV (2015) Exposure of Extensively Farmed Wild Boars (Sus Scrofa Scrofa) to Selected Pig Pathogens in Greece. *Veterinary Quarterly, 35*(2), 97-101. doi:10.1080/01652176.2015.1022666
50. Martínez-Bautista, NR, Sciutto-Conde, E, Cervantes-Torres, J, Segura-Velázquez, R, Mercado García, MC, Ramírez-Mendoza, H, Trujillo Ortega, ME, Delgadillo Alvarez, J, Castillo-Juárez, H, Sanchez-Betancourt, JI (2018) Phylogenetic Analysis of Orf5 and Orf7 of Porcine Reproductive and Respiratory Syndrome (Prrs) Virus and the Frequency of Wild-Type Prrs Virus in México. *Transboundary and Emerging Diseases, 65*(4), 993-1008. doi:10.1111/tbed.12831
51. McGregor, GF, Gottschalk, M, Godson, DL, Wilkins, W, Bollinger, TK (2015) Disease Risks Associated with Free-Ranging Wild Boar in Saskatchewan. *Canadian Veterinary Journal, 56*(8), 839-844.
52. Meemken, D, Tangemann, AH, Meermeier, D, Gundlach, S, Mischok, D, Greiner, M, Klein, G, Blaha, T (2014) Establishment of Serological Herd Profiles for Zoonoses and Production Diseases in Pigs by "Meat Juice Multi-Serology". *Preventive Veterinary Medicine, 113*(4), 589-598. doi:10.1016/j.prevetmed.2013.12.006
53. Mejía Silva, W, Calatayud, D, Zapata, D, Quintero Moreno, A, Torres, P, Chango, M (2012) Seroprevalencia De La Enfermedad De Aujeszky Y Del Síndrome Respiratorio Y Reproductivo Porcino (Prrs) En Granjas Porcinas Del Municipio Mauroa Del Estado Falcón. *Revista Científica, 22*, 139-144.
54. Meléndez, JAS, Arias, JL, Andrade, HF, Ramírez, RÁ (2008) Presencia De Animales Seropositivos Al Síndrome Reproductivo Y Respiratorio Porcino En Nuevo León. *Veterinaria México, 39*(2), 215-221.
55. Meléndez, R, Guzmán, M, Jiménez, C, Piche, M, Jiménez, E, León, B, Cordero, JM, Ramirez-Carvajal, L, Uribe, A, Van Nes, A, Stegeman, A, Vernooij, H, Romero-Zúñiga, JJ (2021) Seroprevalence of Porcine Reproductive and Respiratory Syndrome Virus on Swine Farms in a Tropical Country of the Middle Americas: The Case of Costa Rica. *Tropical Animal Health and Production, 53*(4), 441. doi:10.1007/s11250-021-02799-9

56. Monger, VR, Stegeman, JA, Koop, G, Dukpa, K, Tenzin, T, Loeffen, WLA (2014) Seroprevalence and Associated Risk Factors of Important Pig Viral Diseases in Bhutan. *Preventive Veterinary Medicine, 117*(1), 222-232. doi:10.1016/j.prevetmed.2014.07.005
57. Montagnaro, S, Sasso, S, De Martino, L, Longo, M, Iovane, V, Ghiurmino, G, Pisanelli, G, Nava, D, Baldi, L, Pagnini, U (2010) Prevalence of Antibodies to Selected Viral and Bacterial Pathogens in Wild Boar (Sus Scrofa) in Campania Region, Italy. *Journal of Wildlife Diseases, 46*(1), 316-319. doi:10.7589/0090-3558-46.1.316
58. Mukherjee, P, Karam, A, Singh, U, Chakraborty, AK, Huidrom, S, Sen, A, Sharma, I (2018) Seroprevalence of Selected Viral Pathogens in Pigs Reared in Organized Farms of Meghalaya from 2014 to 16. *Veterinary World, 11*(1), 42-47. doi:10.14202/vetworld.2018.42-47
59. Nemes, I, Molnár, T, Abonyi, T, Terjék, Z, Bálint, Á, Szabó, I (2019) Eradication of Prrs from Backyard Swine Herds in Hungary between 2012 and 2018. *Acta Veterinaria Hungarica, 67*(4), 543-552. doi:10.1556/004.2019.053
60. Oba, P, Wieland, B, Mwiine, FN, Erume, J, Dione, MM (2023) Co-Infections of Respiratory Pathogens and Gastrointestinal Parasites in Smallholder Pig Production Systems in Uganda. *Parasitology Research, 122*(4), 953-962. doi:10.1007/s00436-023-07797-4
61. Pedersen, K, Miller, RS, Musante, AR (2018) Antibody Evidence of Porcine Reproductive and Respiratory Syndrome Virus Detected in Sera Collected from Feral Swine (Sus Scrofa) across the United States. *JSHAP, 26*(1), 41-44.
62. Pérez-Rivera, CM, López, MS, Arnaud-Franco, G, Carreón-Nápoles, R (2017) Detection of Antibodies against Pathogens in Feral and Domestic Pigs (Sus Scrofa) at the Sierra La Laguna Biosphere Reserve, Mexico. *Veterinaria México, 4*(1). doi:10.22201/fmvz.24486760e.2017.378
63. Powell, LF, Cheney, TEA, Williamson, S, Guy, E, Smith, RP, Davies, RH (2016) A Prevalence Study of Salmonella Spp., Yersinia Spp., Toxoplasma Gondii and Porcine Reproductive and Respiratory Syndrome Virus in Uk Pigs at Slaughter. *Epidemiology and Infection, 144*(7), 1538-1549. doi:10.1017/S0950268815002794
64. Prajapati, M, Acharya, MP, Yadav, P, Frossard, J-P (2023) Farm Characteristics and Sero-Prevalence of Porcine Reproductive and Respiratory Syndrome Virus (Prrsv) Antibodies in Pigs of Nepal. *Veterinary Medicine and Science, 9*(1), 174-180. doi:10.1002/vms3.1011
65. Quevedo, V, M., Mantilla, S, J., Portilla, J, K., Villacaqui, A, R., Rivera, G, H. (2018) Seroprevalencia Del Virus Del Síndrome Reproductivo Y Respiratorio Porcino En Cerdos De Crianza No Tecnificada Del Perú. *Revista De Investigaciones Veterinarias Del Perú, 29*(2), 643-651.
66. Rodríguez-Prieto, V, Kukielka, D, Martínez-López, B, de las Heras, AI, Barasona, JÁ, Gortázar, C, Sánchez-Vizcaíno, JM, Vicente, J (2013) Porcine Reproductive and Respiratory Syndrome (Prrs) Virus in Wild Boar and Iberian Pigs in South-Central Spain. *European Journal of Wildlife Research, 59*(6), 859-867. doi:10.1007/s10344-013-0739-2
67. Roic, B, Jemersic, L, Terzic, S, Keros, T, Balatinec, J, Florijancic, T (2012) Prevalence of Antibodies to Selected Viral Pathogens in Wild Boars (Sus Scrofa) in Croatia in 2005–06 and 2009–10. *Journal of Wildlife Diseases, 48*(1), 131-137. doi:10.7589/0090-3558-48.1.131
68. Ruiz-Fons, F, Vicente, J, Vidal, D, Höfle, U, Villanúa, D, Gauss, C, Segalés, J, Almería, S, Montoro, V, Gortázar, C (2006) Seroprevalence of Six Reproductive Pathogens in European Wild Boar (Sus Scrofa) from Spain: The Effect on Wild Boar Female Reproductive Performance. *Theriogenology, 65*(4), 731-743. doi:10.1016/j.theriogenology.2005.07.001
69. Saliki, JT, Rodgers, SJ, Eskew, G (1998) Serosurvey of Selected Viral and Bacterial Diseases in Wild Swine from Oklahoma. *Journal of Wildlife Diseases, 34*(4), 834-838. doi:10.7589/0090-3558-34.4.834
70. Sattler, T, Sailer, E, Wodak, E, Schmoll, F (2012) Serological Detection of Emerging Viral Infections in Wild Boars from Different Hunting Regions of Southern Germany. *Tierarztl Prax Ausg G Grosstiere Nutztiere, 40*(1), 27-32.

71. Sharma, BK, Manandhar, S, Devleesschauwer, B (2016) Serological Evidence of Type 2 (North American Genotype) Porcine Reproductive and Respiratory Syndrome Virus in Nepal. *Tropical Animal Health and Production, 48*(3), 663-666. doi:10.1007/s11250-015-0986-1
72. Siengsanan-Lamont, J, Tum, S, Kong, L, Selleck, PW, Gleeson, LJ, Blacksell, SD (2022) Abattoir-Based Serological Surveillance for Transboundary and Zoonotic Diseases in Cattle and Swine in Cambodia: A Pilot Study in Phnom Penh Province During 2019 and 2020. *Tropical Animal Health and Production, 54*(5), 316. doi:10.1007/s11250-022-03309-1
73. Sookhoo, JRV, Brown-Jordan, A, Blake, L, Holder, RB, Brookes, SM, Essen, S, Carrington, CVF, Brown, IH, Oura, CAL (2017) Seroprevalence of Economically Important Viral Pathogens in Swine Populations of Trinidad and Tobago, West Indies. *Tropical Animal Health and Production, 49*(6), 1117-1124. doi:10.1007/s11250-017-1299-3
74. Stankevicius, A, Buitkuviene, J, Deltuvytiene, J, Cepuliene, R, Zilionyte, V, Pampariene, I, Zymantiene, J (2014) Five Years Seroprevalence Study of Porcine Reproductive and Respiratory Syndrome Virus in Lithuanian Pig and Wild Boar Populations. *Bulletin-Veterinary Institute in Pulawy, 58*, 379-383. doi:10.2478/bvip-2014-0059
75. Štukelj, M, Toplak, I, Vengušt, G (2014) Prevalence of Antibodies against Selected Pathogens in Wild Boars (Sus Scrofa) in Slovenia. *Slovenian Veterinary Research, 51*(1), 21-28.
76. Touloudi, A, Valiakos, G, Athanasiou, LV, Birtsas, P, Giannakopoulos, A, Papaspyropoulos, K, Kalaitzis, C, Sokos, C, Tsokana, CN, Spyrou, V, Petrovska, L, Billinis, C (2015) A Serosurvey for Selected Pathogens in Greek European Wild Boar. *Veterinary Record Open, 2*(2), e000077. doi:10.1136/vetreco-2014-000077
77. Tummaruk, P, Tantilertcharoen, R (2012) Seroprevalence of Porcine Reproductive and Respiratory Syndrome, Aujeszky's Disease, and Porcine Parvovirus in Replacement Gilts in Thailand. *Tropical Animal Health and Production, 44*(5), 983-989. doi:10.1007/s11250-011-9999-6
78. Vengust, G, Valencak, Z, Bidovec, A (2006) A Serological Survey of Selected Pathogens in Wild Boar in Slovenia. *Journal of Veterinary Medicine, Series B, 53*(1), 24-27. doi:10.1111/j.1439-0450.2006.00899.x
79. Vicente, J, León-Vizcaíno, L, Gortázar, C, Cubero, MJ, González, M, Martín-Atance, P (2002) Antibodies to Selected Viral and Bacterial Pathogens in European Wild Boars from Southcentral Spain. *Journal of Wildlife Diseases, 38*(3), 649-652. doi:10.7589/0090-3558-38.3.649
80. Wayne, SR, Morrison, RB, Odland, CA, Davies, PR (2012) Potential Role of Noncommercial Swine Populations in the Epidemiology and Control of Porcine Reproductive and Respiratory Syndrome Virus. *Journal of the American Veterinary Medical Association, 240*(7), 876-882. doi:10.2460/javma.240.7.876
81. Wiratsudakul, A, Prompiram, P, Poltep, K, Tantawet, S, Suraruangchai, D, Sedwisai, P, Sangkachai, N, Ratanakorn, P (2013) A Cross-Sectional Study of Porcine Reproductive and Respiratory Syndrome Virus and Mycoplasma Hyopneumoniae in Wild Boars Reared in Different Types of Captive Setting in Thailand. *Journal of Veterinary Science & Technology, 04*. doi:10.4172/2157-7579.1000146
82. Wu, N, Abril, C, Hinić, V, Brodard, I, Thür, B, Fattebert, J, Hüssy, D, Ryser-Degiorgis, MP (2011) Free-Ranging Wild Boar: A Disease Threat to Domestic Pigs in Switzerland? *Journal of Wildlife Diseases, 47*(4), 868-879. doi:10.7589/0090-3558-47.4.868
83. Wyckoff, AC, Henke, SE, Campbell, TA, Hewitt, DG, VerCauteren, KC (2009) Feral Swine Contact with Domestic Swine: A Serologic Survey and Assessment of Potential for Disease Transmission. *Journal of Wildlife Diseases, 45*(2), 422-429. doi:10.7589/0090-3558-45.2.422
84. Yu, H, Zhang, L, Cai, Y, Hao, Z, Luo, Z, Peng, T, Liu, L, Wang, N, Wang, G, Deng, Z, Zhan, Y (2022) Seroprevalence of Antibodies to Classical Swine Fever Virus and Porcine Reproductive and Respiratory Syndrome Virus in Healthy Pigs in Hunan Province, China. *Polish Journal of Veterinary Sciences, 25*(3), 375-381. doi:10.24425/pjvs.2022.142020

85. Zhao, P, Wang, C, Cao, W, Fang, R, Zhao, J (2022) Risk Factors and Spatial-Temporal Analysis of Porcine Reproductive and Respiratory Syndrome Seroprevalence in China before and after African Swine Fever Outbreak. *Frontiers in Veterinary Science, 9*, 929596. doi:10.3389/fvets.2022.929596
86. Zupancić, Z, Jukić, B, Lojkić, M, Cac, Z, Jemersić, L, Staresina, V (2002) Prevalence of Antibodies to Classical Swine Fever, Aujeszky's Disease, Porcine Reproductive and Respiratory Syndrome, and Bovine Viral Diarrhoea Viruses in Wild Boars in Croatia. *Journal of Veterinary Medicine Series B-Infectious Diseases and Veterinary Public Health, 49*(5), 253-256. doi:10.1046/j.1439-0450.2002.00562.x
