# Supplementary material for: A Systematic Review and Meta-Analysis on the Global Seroprevalence of Porcine Reproductive and Respiratory Virus (PRRSV) in Pigs and Wild Boars: A Widespread and Impactful Swine Virus
Source: Vet Sci. 2026 Mar 23;13(3):304. doi: 10.3390/vetsci13030304 (PMC13030171; doi:10.3390/vetsci13030304)
Supplement: Supplementary file 1 [file vetsci-13-00304-s001.zip › Supplementary_Material_4.pdf]

Supplementary Material S4: Sub-group analyses of eligible PRSSV serological studies.

Sub-group analyses of eligible PRSSV serological studies according to the criteria applied for the systematic revision of the literature. The analyses were independently performed considering the geographical distribution of the studies, the species sampled, the serological method used and the decade when the study took place.

Table S1. Sub-group analyses of PRSSV serological studies deemed eligible according to the criteria applied for the systematic revision of the literature. The analyses were independently performed considering the geographical distribution of the studies, the species sampled, the serological method used and the decade when the study took place.

Subgroup	No of prevalence inputs	Sample size	Proportion	95% CI	Tau²	Difference between groups
**Geographical distribution**						**p<0.0001**
Asia	24	33,824	0.29	0.16 – 0.43	0.13	
Europe	35	60,253	0.09	0.04 – 0.15	0.1	
Americas	20	39,068	0.09	0.03 – 0.18	0.13	
Africa	3	1,149	0.18	0 – 0.93	0.12	
Australia	1	875	0.005	0 – 0.01	--	
**Species**						**p<0.0001**
Pigs	53*	114,665	0.26	0.18 – 0.35	0.11	
Wild boars	37*	20,504	0.02	0.01 - 0.03	0.01	
**Serological method used**						p=0.28
ELISA	77	127,881	0.12	0.08 – 0.18	0.11	
IFAT	3	6,358	0.31	0 – 0.86	0.45	
IPMA	2	730	0.23	0 – 1.0	0.05	
GICA	1	200	0.18	0.13 – 0.24	--	
**Decade**						**p=0.02**
I (1993-2003)	23	22,598	0.15	0.05 – 0.30	0.13	
II (2004-2014)	40	77,655	0.08	0.04 – 0.13	0.08	
III (2015-2024)	20	34,916	0.25	0.13 – 0.40	0.13	

*Studies that included both wild boars and domestic pigs were added, as duplicates, to the respective category.

Table S2. Sub-group analysis of PRSSV serological studies deemed eligible according to the criteria applied for the systematic revision of the literature. The housing system, consistently avaibale for wild boars only, was considered. .

Subgroup (species-specific dataset)	No of prevalence inputs	Sample size	Proportion	95% CI	Tau²	Difference between groups
**Housing (Wild boars)**						p=0.98
Free-range	31	13,923	0.02	0.01 – 0.05	0.02	
Fenced	5	1,002	0.02	0 – 0.1	0.001	
